# Discovery of novel antibacterial agent for the infected wound treatment: all-hydrocarbon stapling optimization of LL-37

**DOI:** 10.7150/thno.87916

**Published:** 2024-01-20

**Authors:** Yanan Zhang, Mengjun Zheng, Zhe Wang, Zhinan Liu, Sumeng Chen, Xiang Li, Yejiao Shi, Honggang Hu

**Affiliations:** 1 Institute of Translational Medicine, Shanghai University, Shanghai 200444, China.; 2 School of Medicine, Shanghai University, Shanghai 200444, China.; 3 School of Pharmacy, Naval Medical University, Shanghai 200433, China.; 4 Institute of Bioengineering, College of Chemical and Biological Engineering, Zhejiang University, Hangzhou, 310027, China.

**Keywords:** LL-37, all-hydrocarbon stapling, antibacterial, anti-inflammatory, infected wound healing

## Abstract

**Rationale:** Antimicrobial peptide LL-37 has been recognized as a favorable alternative to antibiotics due to its broad antibacterial spectrum, low resistance development and diverse biological activities. However, its high manufactory cost, poor proteolytic stability, and unpredictable cytotoxicity seriously hindered its medical translation.

**Methods:** To push the frontiers of its clinical application, all-hydrocarbon stapling strategy was exploited here for the structural modification of KR-12, the core and minimal fragment of LL-37.

**Results:** Based on a library of KR-12 derivatives that designed and synthesized to be stapled at positions of either *i, i+4* or *i, i+7*, structure to activity relationship was investigated. Among them, KR-12(Q_5_, D_9_) with the glutamine and aspartic acid residues stapled displayed increased helical content and positive charge. The reinforced α-helical conformation not only protected it from proteolytic hydrolysis but also improved its antibacterial efficacy via effective membrane perturbation and anti-inflammatory efficacy via compact LPS binding. Besides, the increased positive charge endowed it with an enhanced therapeutic index. On infected wound mouse model, it was demonstrated to eliminate bacteria and promote wound closure and regeneration effectively.

**Conclusion:** Overall, the all-hydrocarbon stapling was proven to lay the foundation for the future development of antibacterial agents. KR-12(Q_5_, D_9_) could serve as a lead compound for the clinical treatment of bacterial infections.

## Introduction

Antibiotics are one of the most effective drugs for fighting life-threatening bacterial infections in human history. Their discovery has transformed the modern medicine and started the golden era for the treatment of previously fatal infectious diseases 1-3. However, due to their overuse and misuse in humans and animals, bacterial tolerance and resistance have emerged, compromising their therapeutic efficacy 4, 5. According to a review commissioned by the United Kingdom government, 10 million people will die every year by 2050, unless a global response to the antimicrobial resistance is mounted 6. Although new antibiotics can be discovered via the high-throughput screening of chemical libraries and synthesized via the traditional approaches of medicinal chemistry, the pace at which they are brought to market and delivered to patients is too slow to meet the challenges posed by resistant bacteria 7-9. The escalating crisis of bacterial infections has led to urgent calls for the discovery of novel antibacterial agents globally.

Antimicrobial peptides (AMPs) have been considered as an alternative therapeutic agent to conventional antibiotics 10, 11. These naturally occurring peptides, typically consisting of less than 100 hydrophobic and cationic amino acids in a certain sequence combination, exert their antibacterial effects mainly by disrupting the integrity of bacterial cell membranes 12. Therefore, they are unlikely to develop resistance compared to the conventional antibiotics 13, 14. Among them, LL-37 is the only cathelicidin antimicrobial peptide found existing in humans 15, 16. It consists of 37 amino acids presenting in an amphipathic α-helical structure 17. The positive charge of LL-37 facilitates its initial adsorption onto the negatively charged bacterial cell membrane. Whereas the amphipathic α-helical structure of LL-37 allows its insertion into the phospholipid bilayers of the bacterial cell membrane, destroying the fluidity and forming ion channels of the phospholipid bilayers, which ultimately leads to the bacterial content leakage and cell death 18. Therefore, LL-37 has a broad-spectrum antibacterial activity. In addition, LL-37 also exhibits the ability to modulate the inflammatory response and counteract sepsis by binding to lipopolysaccharide (LPS) and lipoteichoic acid (LTA) specifically, acting as an endotoxin neutralizing agent 19-21. Besides, it is involved in many other key biological processes such as angiogenesis 22, 23, re-epithelialization 24, 25, as well as wound closure 26, 27. However, despite its diverse bioactivities, the clinical application of LL-37 is hindered by high manufactory cost due to its long sequence, poor *in vivo* stability against protease hydrolysis, and unpredictable cytotoxicity derived from its positive charge 28.

The limitations of LL-37 have promoted the research on the identification of its bioactive regions. It has been truncated into number of short fragments to dissect the sequence-activity relationship 29. Many active fragments were found located at the *C*-terminal domain of LL-37, specifically residue 17-32 30, 31. The minimal sequence with antibacterial activity, comprising residue 18-29 (KRIVQRIKDFLR), was then identified and referred to as KR-12 32, 33. It displayed similar antibacterial potency as LL-37 but lower cytotoxicity, making it an attractive lead peptide for the future development of antibacterial agents 15. However, KR-12 has an inevitable disadvantage as a linear peptide, in terms of insufficient antibacterial activity and poor proteolytic stability, to meet the clinical demands. Therefore, it is of great significance to perform structural modifications of KR-12, improving its antibacterial potency and enhancing its proteolytic resistance.

To release the potential medical use of linear peptides, diverse modification strategies, including lipidation, glycosylation and cyclization have been proposed to engineer selective, stable and potent peptide therapeutics 11, 34-40. Among them, the all-hydrocarbon stapling strategy is an efficient method to stabilize the preorganized helical conformation of the peptide with a reduced entropic penalty. It uses Grubbs metathesis catalysts to cross-linking either side chain to the terminus or side chain to side chain of two non-natural amino acids at the positions of either *i, i+4* or *i, i+7* of linear peptides 41-43. Our previous studies have successfully demonstrated the all-hydrocarbon stapling strategy is capable of reinforcing the native α-helical conformation of peptides, thereby protecting peptides from proteolytic hydrolysis and improving its biological activities ranging from antitumor to antimicrobial 44-48. Stapled antimicrobial peptides, such as Magainin II and Anoplin, were emerging as promising antimicrobial agents, enabling enhanced structure stability and antimicrobial potency 9, 49. Derived from cecropin-melittin, L8 peptide with a very similar sequence (KRIVQRIKKWLR) as KR-12 have also been reported to possess improved proteolytic stability and *in vivo* antibacterial activity on a zebrafish embryo infection model 50. Despite all that, the stapling modification of KR-12 has never been performed.

Herein, the present study aims to push the frontier of KR-12 optimization by exploiting the all-hydrocarbon stapling strategy. A library of KR-12 derivatives that stapled at the positions of *i, i+4* and *i, i+7* were designed and synthesized. Their antibacterial activities and physicochemical parameters were assessed and compared to those of KR-12 and LL-37. Based on antibacterial activity, proteolytic resistance and hemolytic activities, the outstanding candidate was identified, with its structure-to-activity relationship being discussed. In addition, its antibacterial and anti-inflammatory mechanisms were initially explored. Its potential as a promising peptide lead, in terms of safety and efficacy, were also evaluated *in vitro* and *in vivo* on infected wound model in the end for developing an antibacterial agent against bacterial infections.

## Results and Discussions

### Design and synthesis of the stapled KR-12 derivatives

**Figure 1:**
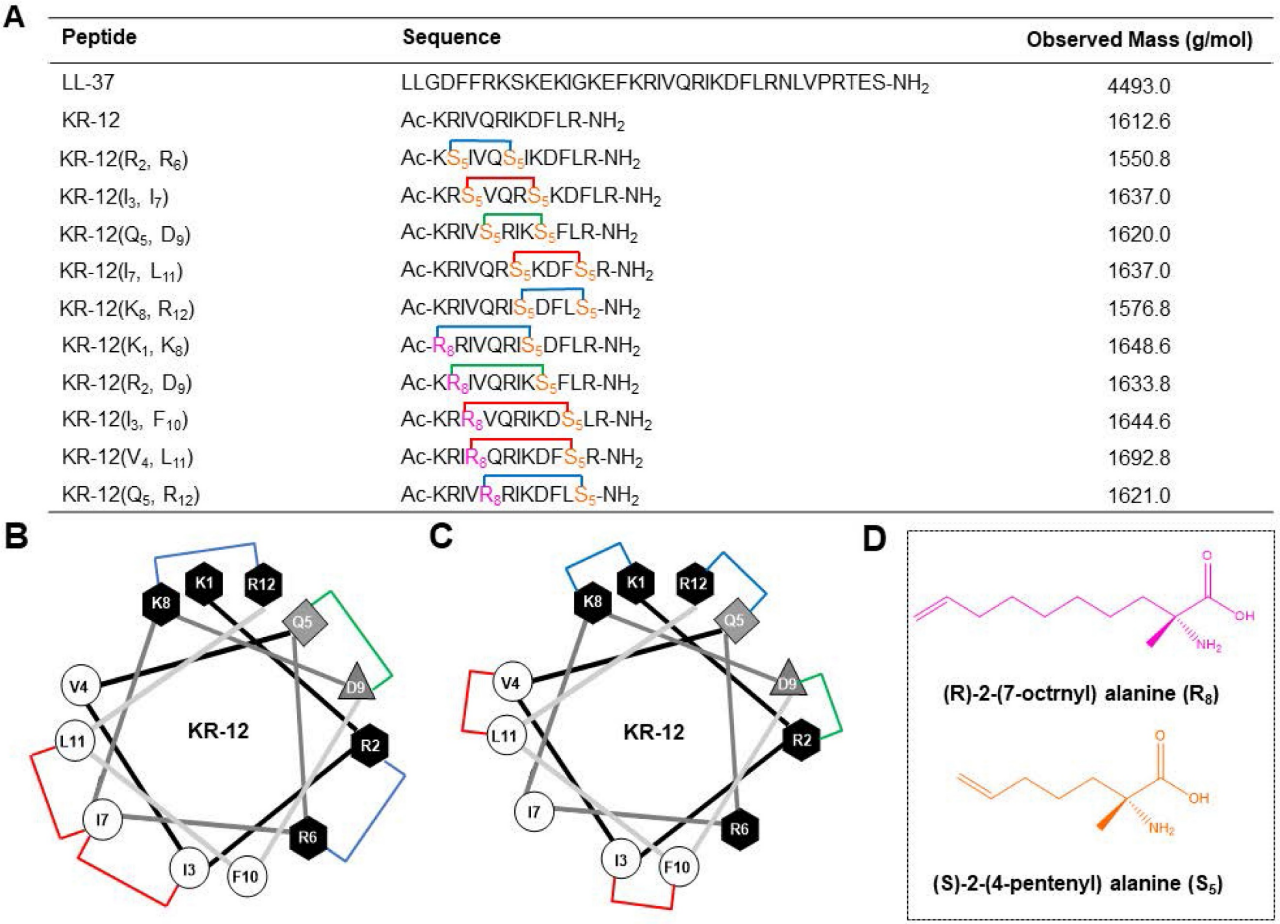
(**A**) Sequence and observed mass of the LL-37, KR-12 and its stapled derivatives that designed in the present study. (**B**) Helical wheel diagram of KR-12 and its (*i, i + 4*) stapled derivatives. The stapled positions are indicated by colored lines. (**C**) Helical wheel diagram of KR-12 and its (*i, i + 7*) stapled derivatives. The stapled positions are indicated by colored lines. (**D**) Chemical structure of the (R)-2-(7-octenyl) alanine (R_8_) and (S)-2-(4-pentenyl) alanine (S_5_) that cross-linked by ring-closing metathesis.

Based on a previous alanine scanning study, which has demonstrated the important role of hydrophobic and cationic residues in the antibacterial efficacy of KR-12 32, five KR-12 derivatives that stapled at the (*i, i+4*) positions were initially designed (**Figure 1A**). As displayed in **Figure 1B**, hydrophobic amino acids at the positions of (*3, 7*) and (*7, 11*), cationic amino acids at the positions of (*2, 6*) and (*8, 12*), as well as anionic amino acids at the positions of (*5, 9*) were substituted with (S)-2-(4-pentenyl) alanine (S_5_) for the one helical turn stapling. As for the two helical turn stapling, five more KR-12 derivatives were designed (**Figure 1A**), with amino acids at their positions of *i, i+7* (**Figure 1C**) being replaced by (R)-2-(7-octenyl) alanine (R_8_) and (S)-2-(4-pentenyl) alanine (S_5_) (**Figure 1D**), respectively.

**Scheme 1:**
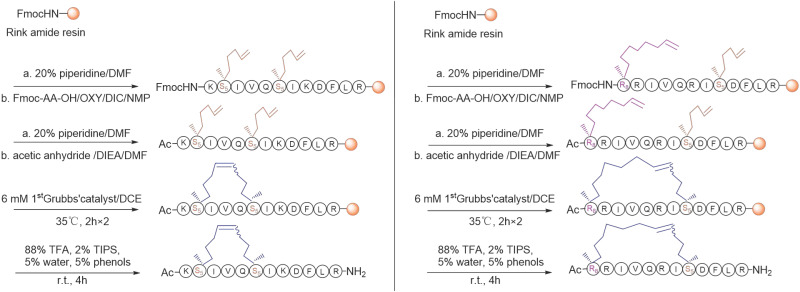
The synthetic route of stapled KR-12 derivatives.

For the synthesis, linear peptide precursors with the two-terminal olefin anchored residues were firstly prepared via the conventional solid-phase peptide synthesis and then stapled via the ring-closing olefin metathesis, which was catalyzed by the first-generation Grubbs' regent (**Scheme 1**) 43, 51. After cleaved from the rink amide resin, the crude peptides were purified by the preparative RP-HPLC. Due to olefin recombination, the (*i, i+7*) stapled derivatives are theoretically prone to exist as two conformational isomers 52. In the present study, only one conformational isomerism of each derivative, which was eluted firstly from the RP-HPLC, was separated, and investigated in the study. All the designed KR-12 derivatives were successfully obtained at a high yield. Their purity and mass were confirmed by analytical RP-HPLC and ESI-MS, respectively (**Figure S1-S12**).

### Antibacterial activity

According to the protocol of Clinical & Laboratory Standards Institute, the minimum inhibitory concentration (MIC) of the linear and stapled peptides against a panel of gram-positive and gram-negative bacteria was determined by the broth microdilution method. As displayed in **Table 1**, compared to LL-37, the linear KR-12 exhibited a 4-fold enhanced antibacterial efficacy against the gram-positive *S. aureu*s but similar efficacy against the gram-positive *S. epidermidis* and *E. faecalis*, as well as the gram-negative bacteria. Upon the all-hydrocarbon stapling modification, KR-12(Q_5_, D_9_) displayed the most significantly improved antibacterial activities, with 16-fold and 4-fold reduced MIC than the linear KR-12 being determined against* S. epidermidis, E. faecalis*,* E. coli* and *A. baumannii,* as well as *S. aureus* and *P. aeruginosa*, respectively. In comparison, KR-12(I_3_, I_7_), KR-12(I_7_, L_11_), KR-12(I_3_, F_10_) and KR-12(V_4_, L_11_) showed moderately improved antibacterial efficacy with 2-4-fold reduced MIC against gram-negative bacteria. However, as for gram-positive bacteria, only KR-12(I_3_, I_7_) and KR-12(I_7_, L_11_) with one helical turn stapling exhibited enhanced antibacterial efficacy, while KR-12(I_3_, F_10_) and KR-12(V_4_, L_11_) with two helical turn stapling did not. Similarly, almost the same trend of change in antibacterial efficacy were demonstrated by KR-12(R_2_, D_9_) and KR-12(Q_5_, R_12_) as KR-12(I_3_, F_10_) and KR-12(V_4_, L_11_). By contrast, attenuated antibacterial potency was observed for KR-12(R_2_, R_6_), KR-12(K_8_, R_12_) and KR-12(K_1_, K_8_), except for the slightly increased antibacterial efficacy against gram-positive *S. aureus, S. epidermidis* and* E. faecalis*, as well as gram-negative *E. coli* and* A. baumannii* being observed for KR-12(K_8_, R_12_).

Overall, KR-12 derivatives that stapled at the positions of *i, i+4* exhibited comparatively improved antibacterial activities than the derivatives stapled at the positions of *i, i+7*. Therefore, the (*i, i+4*) position modified derivatives were mainly analyzed and discussed in the subsequent study, with emphasis on KR-12(Q_5_, D_9_), whose antibacterial efficacy is the most potent.

### Structure activity relationship

**Figure 2:**
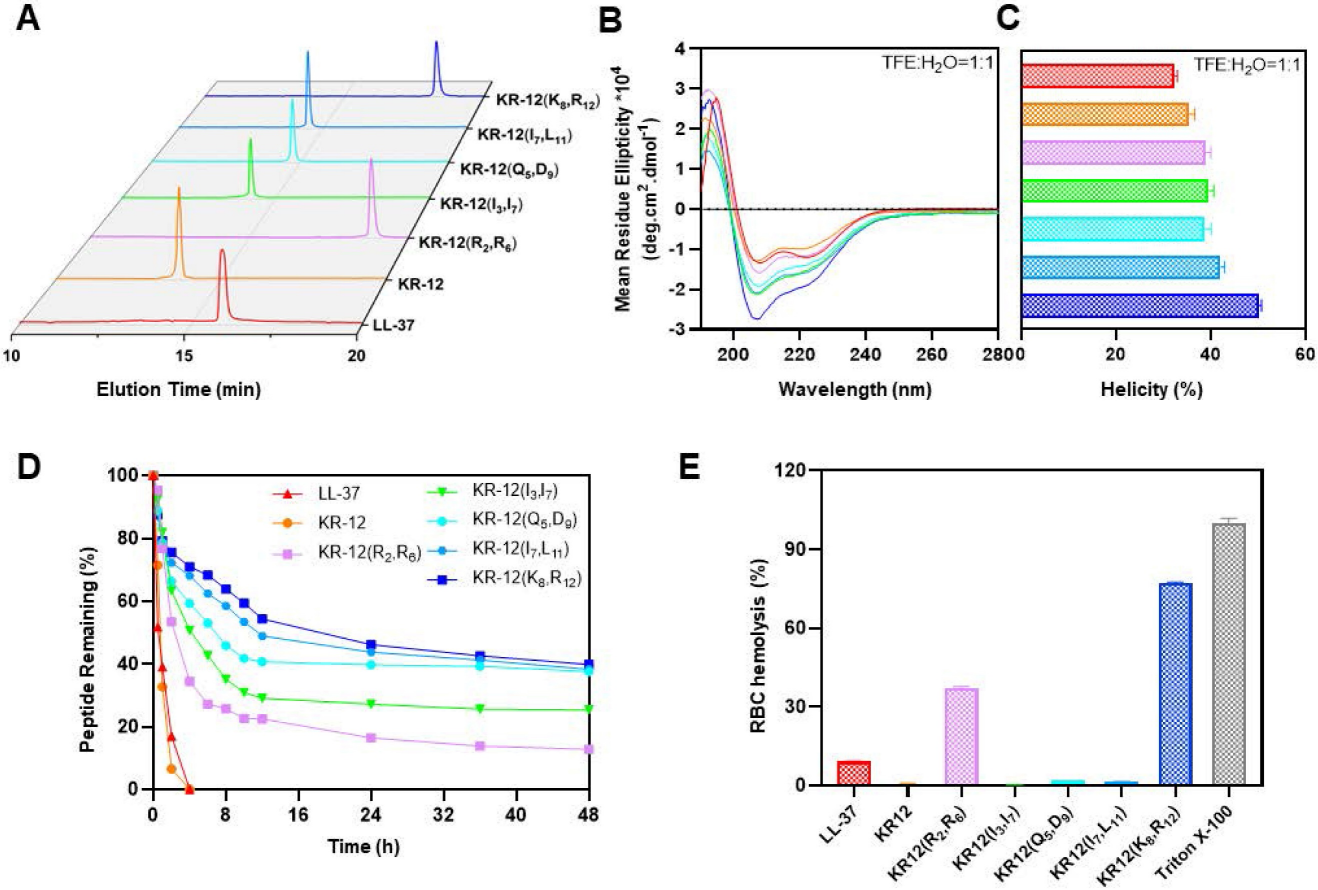
Structural parameters, serum stability and hemolysis of LL-37, KR-12 and its stapled derivatives: (**A**) RP-HPLC spectra indicating the hydrophobicity, the vertical axis is the absorbance of peptides at the wavelength of 220 nm; (**B**) CD spectra indicating the α-helicity; (**C**) α-helicity calculated from the CD spectra; (**D**) Serum stability in presence of human serum for 48 h; (**E**) Hemolysis percentage of rabbit erythrocyte in presence of 256 μg/mL LL-37, KR-12 and its derivatives.

To uncover the structure-activity relationship of KR-12 and its stapled derivatives, their physicochemical properties in terms of hydrophobicity, net charge and α-helicity were investigated (**Table 2**). As shown in **Figure 2A**, the hydrophobicity was measured by retention time obtained from the spectra of RP-HPLC. Upon the stapling modification, all the KR-12 derivatives exhibited increased hydrophobicity as longer retention times were observed. However, the increased hydrophobicity did not seem to facilitate the improvement of antibacterial efficacy. Because among the five (*i, i+4*) position stapled derivatives, the most hydrophobic KR-12(R_2_, R_6_) displayed the least antibacterial efficacy.

As for net charge at pH 7.4, only KR-12(I_3_, I_7_) and KR-12(I_7_, L_11_) remain the same as the linear KR-12. KR-12(R_2_, R_6_) and KR-12(K_8_, R_12_) with the positively charged arginine and lysine residues being substituted and stapled present decreased net charge (**Table 2**). While KR-12(Q_5_, D_9_) with the negatively charged aspartic acid being substituted and stapled presents an increased net charge. The increased positive net charge endowed KR-12(Q_5_, D_9_) with strengthened antibacterial capacity. On the contrary, the decreased positive net charge led to a weakened antibacterial capacity for KR-12(R_2_, R_6_) and KR-12(K_8_, R_12_). These results indicated that the positive charge of stapled KR-12 derivatives is positively correlated to their antibacterial capacity.

The α-helicity of stapled KR-12 derivatives was calculated from the circular dichroism spectra as demonstrated in **Figure 2B**. Upon the stapling modification, all the five derivatives displayed increased α-helicity in aqueous solution containing 50% trifluoroethanol (TFE), which can lower the polarity of the solution thus mimic the hydrophobic environment of bacterial membranes (**Figure 2C**). These results are consistent with the finding reported by Schouten et al. that staple modification increased the α-helicity of AMP with a similar sequence of KR-12 50. Specially, compared to KR-12, the α-helicity of the stapled KR-12 derivatives increased gradually with the stapling position of the peptide moving from the *N*-terminus to the *C*-terminus. As a result, KR-12(K_8_, R_12_) exhibited the highest α-helicity, which may contribute to its slightly improved antibacterial efficacy even though its net charge is relatively low.

Overall, the antibacterial activity of KR-12 derivatives could be influenced by their physicochemical parameters upon the stapling modification. According to the amino acid residues that being substituted and stapled, the net charge of derivatives varied and positively correlated to their antibacterial efficacy. In addition, both the hydrophobicity and α-helicity of derivatives increased after stapling. However, only the increased α-helicity is capable of improving the antibacterial activities.

### Serum stability and hemolytic activity

Maintaining high stability and presenting high compatibility in the complex physiological environment are important criterions for the clinical translation of AMPs. Given the improved antibacterial activity of KR-12 derivatives stapled at the (*i, i+4*) positions, their therapeutic potential in terms of serum stability and hemolytic activity was evaluated. The derivatives were mixed with human serum in pH 7.4 PBS, simulating the degradation process of peptides by proteases *in vivo*. The percentages of intact derivatives were monitored and determined with time according to the PR-HPLC. As displayed in **Figure 2D**, the linear LL-37 and KR-12 degraded very rapidly and more than 50% was hydrolyzed within 1 h. By contrast, the stapled derivatives exhibited enhanced resistance against human serum. The time at which 50% of the derivatives degraded was increased gradually from 2.38 h to 18.28 h, with the stapling position of the peptide moving from the* N-* to *C-terminus*. These results were consistent with the findings reported by Schouten et al. that all-hydrocarbon stapling could increase the serum stabilities of AMPs 50. In combination with the trend of change in α-helicity, the gradually increased serum stability of derivatives upon stapling modification may result from their gradually locked conformation, restricting the contact between the serum and the degradation site.

Based on the improved serum stability of the KR-12 derivative, the antibacterial activities in clinically relevant conditions were assessed. LL-37, KR-12 and KR-12(Q_5_, D_9_) with the most potent antibacterial efficacy were selected to incubate with 50% human serum for 4 h before their MIC determination. As shown in **Table S1**, the linear LL-37 and KR-12 lost their antibacterial activities against all bacteria with the pre-incubation of human serum. By contrast, the stapled KR-12(Q_5_, D_9_) maintained its antibacterial activity, confirming its improved stability against human serum.

The hemolytic activity of peptides was tested using rabbit erythrocytes (**Figure S13**). As shown in **Figure 2E**, compared to LL-37 with a hemolysis percentage of 9.2% at 256 μg/mL, KR-12(R_2_, R_6_) and KR-12(K_8_, R_12_) exhibited significantly higher hemolysis percentage at 37.1% and 77.1%, respectively. The preferential disruption capacity of KR-12(R_2_, R_6_) and KR-12(K_8_, R_12_) towards mammalian cell membranes is probably caused by their increased hydrophobicity and decreased net charge, contributing to their preferred interactions with mammalian cell membranes. By contrast, KR-12 as well as its three other derivatives, namely KR-12(I_3_, I_7_), KR-12(Q_5_, D_9_) and KR-12(I_7_, L_11_), displayed negligible hemolytic activities, suggesting better compatibility towards mammalian cells. These results were consistent with the findings reported by Schouten et al. that significantly reduced hemolysis was observed for an AMP with a similar sequence of KR-12 and stapled at the same position as KR-12(I_3_, I_7_) and KR-12(I_7_, L_11_).

Overall, KR-12(I_3_, I_7_), KR-12(Q_5_, D_9_) and KR-12(I_7_, L_11_) with enhanced antibacterial efficacy displayed improved serum stability and hemolytic activity. Therefore, these three derivatives were mainly focused on in the subsequent investigations.

### Antibacterial mechanism

**Figure 3:**
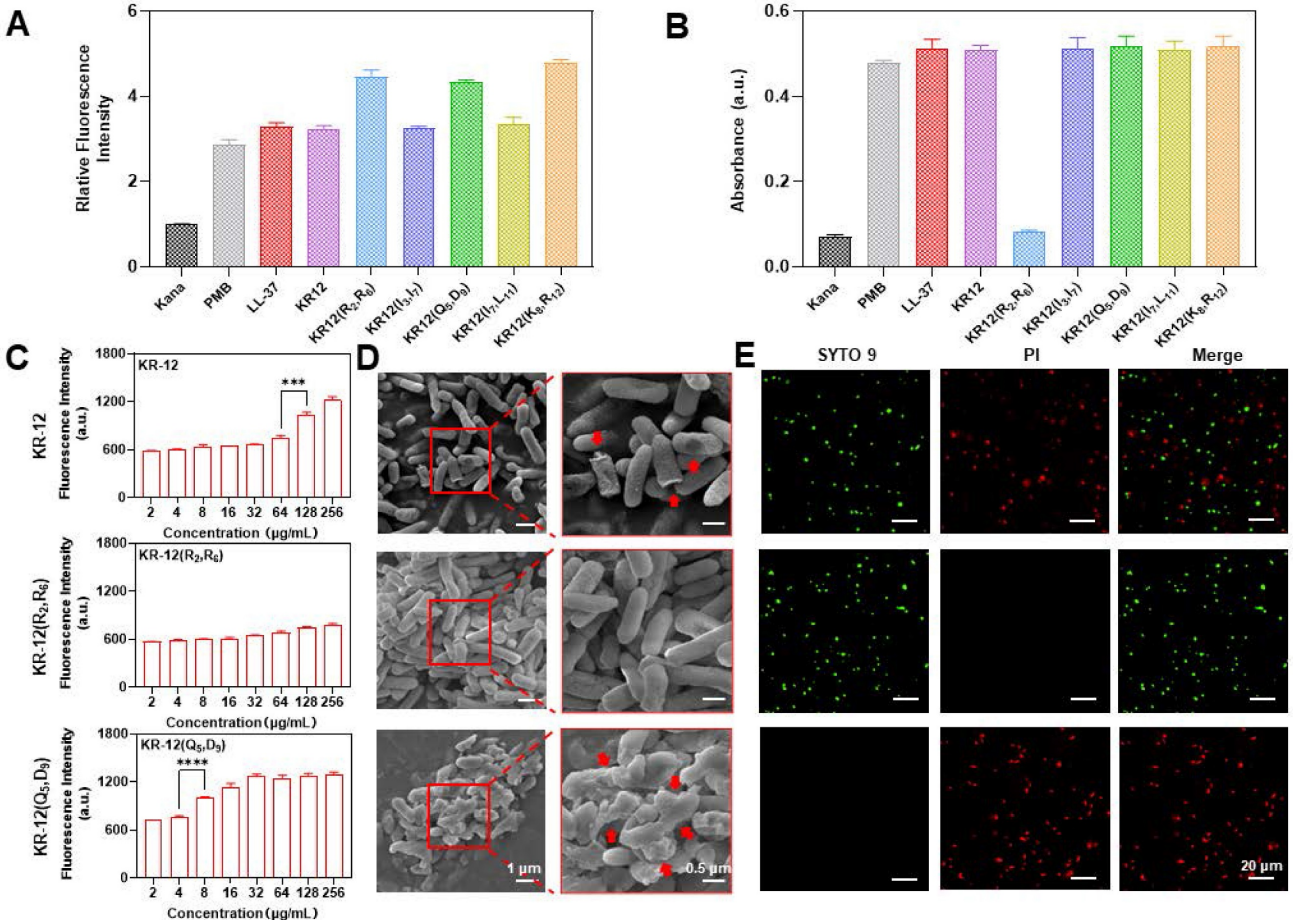
** Antibacterial mechanism of KR-12 and its derivatives on ATCC25922 *E. coli***: (**A**) Outer membrane perturbation assay performed with NPN; (**B**) Inner membrane permeability assay performed with ONPG; (**C**) Inner membrane depolarization assay performed with DiSC_3_-5; (**D**) SEM morphology images of *E. coli* under the treatment of 64 μg/mL KR-12, KR-12(R_2_, R_6_) and KR-12(Q_5_, D_9_) for 24 h; (**E**) Microplate fluorescent images of *E. coli* under the treatment of 64 μg/mL KR-12, KR-12(R_2_, R_6_) and KR-12(Q_5_, D_9_) for 24 h. Viable bacterial cells were stained green by SYTO9, whereas dead bacterial cells were stained red by PI. Statistical significance of differences was determined using an independent sample t-test with *p < 0.05, **p < 0.01, ***p < 0.001, ****p <0.0001.

As most AMPs, LL-37 and KR-12 exert their antibacterial activity via the membrane perturbation mechanism. Thus, whether the KR-12 derivatives retain the same membrane activity upon stapling was examined on the gram-negative *E. coli*. Commercial antibiotic polymyxin b with membrane activity and kanamycin with nonmembrane activity were used as positive and negative controls, respectively. 1-*N*-phenylnaphthyamine (NPN), a hydrophobic fluorescent probe that emit fluorescence once interacts with the hydrophobic region of phospholipid bilayers, was used to evaluate the disturbance of bacterial outer membrane (OM) 53. All the KR-12 derivatives were demonstrated to perturbate the OM in a concentration-dependent manner (**Figure S14**). The perturbative capacity of KR-12 derivatives was found to be positively related to their hydrophobicity as the more hydrophobic KR-12(R_2_, R_6_), KR-12(Q_5_, D_9_) and KR-12(K_8_, R_12_) were observed to cause more severe OM perturbation (**Figure 3A**). In addition, the permeability of bacterial inner membrane (IM) was evaluated utilizing *O*-nitrophenyl-β-D-galactopyranoside (ONPG), which is a colorimetric substrate of the cytoplasmic content β-galactosidase 54. Except for KR-12(R_2_, R_6_), all other KR-12 derivatives were proven to disrupt the IM in a concentration-dependent manner (**Figure S15**). The inactive IM activity of KR-12(R_2_, R_6_) is consistent with its undetected antibacterial activity up to a concentration of 128 μg/mL (**Figure 3B**). The KR-12 and its derivatives induced dysfunction of IM was confirmed with 3,3′-dipropylthiacarbocyanine iodide (DiSC_3_-5), a membrane potential sensitive probe (**Figure S16**) 55. As shown in **Figure 3C**, both KR-12 and KR-12(Q_5_, D_9_) were capable of increasing the fluorescence intensity of DiSC_3_-5 in a concentration-dependent manner. Remarkably, the most notable fluorescent increase occurred at the MIC of KR-12 and KR-12(Q_5_, D_9_), indicating the depolarization and dysfunction of bacterial IM. By contrast, no such membrane activity was observed for KR-12(R_2_, R_6_) with undetected bacterial activity.

To facilitate the direct observation of membrane perturbation, scanning electron microscopy (SEM) was used to inspect the morphology of bacteria with the treatment of 64 μg/mL KR-12, KR-12(R_2_, R_6_) and KR-12(Q_5_, D_9_). As expected, KR-12 induced partial pore formation and membrane rupture compared to the KR-12(R_2_, R_6_) treated *E. coli*, which displayed an intact and smooth surface (**Figure 3D**). In sharp contrast, KR-12(Q_5_, D_9_) resulted in obvious blebbing, irregular collapse and rough debris of *E. coli*. Depending on the different degrees of membrane damage, the viability of *E. coli* varied. As demonstrated in** Figure 3E**, *E. coli* exposed to KR-12 were stained green by SYTO9 and red by propidium iodide (PI) at the same time, indicating the existence of both live and dead bacteria. While the KR-12(R_2_, R_6_) and KR-12(Q_5_, D_9_) incubated *E. coli* appeared only in green or red, suggesting the live or dead status of bacteria, respectively.

Overall, except for KR-12(R_2_, R_6_), all other (*i, i+4*) position stapled KR-12 derivatives exerted their antibacterial activity via the membrane perturbation mechanism as the linear KR-12. Among them, KR-12(Q_5_, D_9_) with the strongest membrane depolarization and dysfunction capacity exhibited the highest antibacterial potency. Whereas no antibacterial activities were observed for KR-12(R_2_, R_6_), which was only able to damage the OM rather than the IM of bacteria.

### Cytotoxicity and therapeutic index

**Figure 4:**
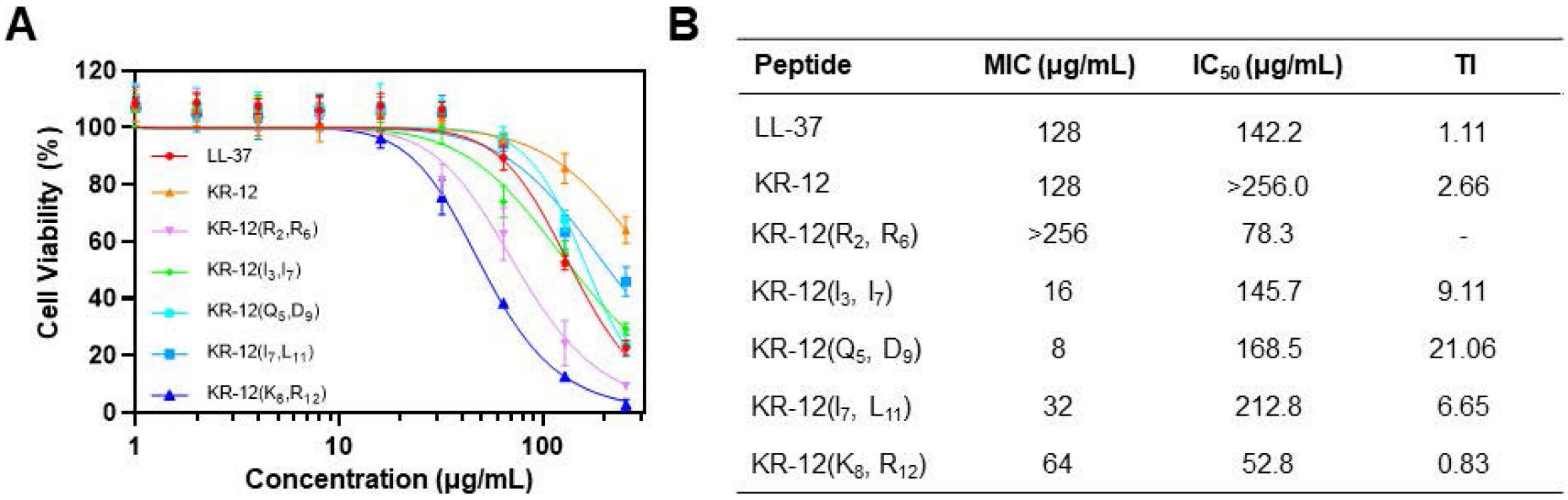
(**A**) Cytotoxicity of LL-37, KR-12 and its stapled derivatives toward the mammalian NIH-3T3 cell; (**B**) Table of the therapeutic index of LL-37, KR-12 and its stapled derivatives determined based on the ratio of their IC_50_ against NIH-3T3 cell to MIC against *E. coli*.

For development of the stapled KR-12 derivatives to treat bacterial infections *in vivo*, their cytotoxicity was examined via the CCK-8 assay, which could provide a quantitative assessment of cell viability based on the dehydrogenase activity. The mouse embryonic fibroblast NIH-3T3 cell was selected and incubated with various concentrations of KR-12 and its stapled derivatives. As shown in** Figure 4A**, KR-12 displayed relatively weak cytotoxic activity towards NIH-3T3, with only a 36% loss of viability determined at the maximum concentration of 256 μg/mL. In comparison, its five stapled derivatives exhibited concentration-dependent cytotoxicity as LL-37. By calculating the IC_50_ of the KR-12 derivatives, it is worth noting that the cytotoxicity of KR-12 derivatives towards NIH-3T3 grew incrementally with the increasing of their hydrophobicity (**Figure 4B**).

To further evaluate the selective toxicity of KR-12 derivatives, their therapeutic index (TI) was determined on the ratio of their IC_50_ to MIC. Thus, a high TI value of the derivative, produced by its high IC_50_ (weak cytotoxic activity) and low MIC (strong antibacterial activity), is a reflection of its great selectivity towards bacterial cells. Except for KR-12(R_2_, R_6_) and KR-12(K_8_, R_12_), all the other three derivatives present enhanced TI upon the stapling modification (**Figure 4B**). Among them, KR-12(Q_5_, D_9_) has the highest TI, which is 19.2 and 8.1 times higher than the values of LL-37 and KR-12, respectively. Therefore, KR-12(Q_5_, D_9_) with the greatest bacterial selectivity demonstrated the most preferred therapeutic potential.

### *In vivo* therapeutic performance

**Figure 5:**
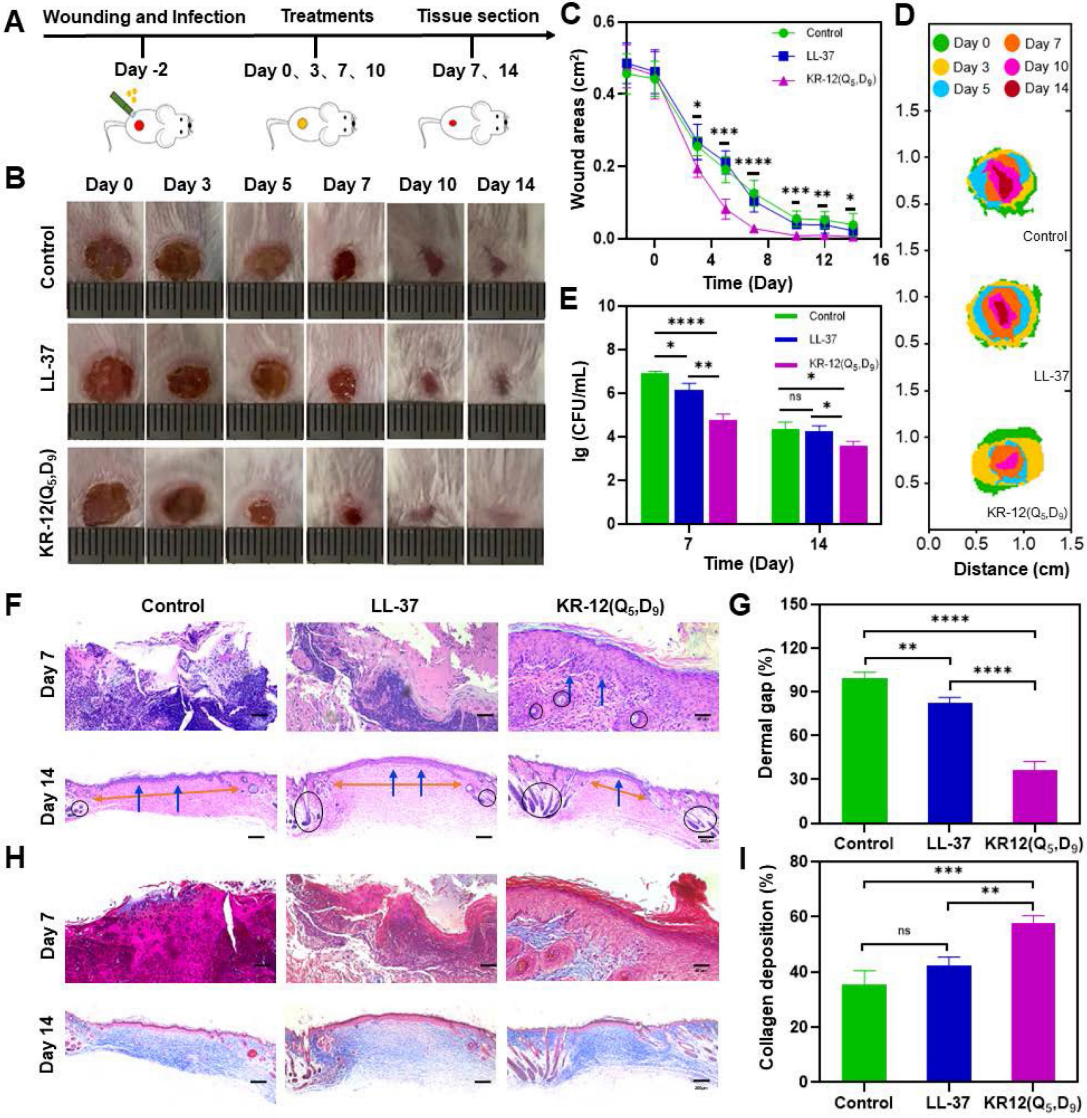
***In vivo* therapeutic performance of LL-37 and KR-12(Q_5_, D_9_) on the mouse model with *E. coli* infected wound**: (**A**) Schematic illustration of wound construction, infection, and treatment procedure; (**B**) Representative images of the infected wound at different time points for each treatment group; (**C**) Statistical graph of wound areas during the 14 days of treatment; (**D**) Simulation analysis of the infected wound healing progression with different treatments; (**E**) CFU of *E. coli* from the infected skin tissues after the different treatments for 7 and 14 days; (**F**) Histological analysis on the infected wound tissue sections stained with H&E staining after 7 and 14 days of different treatments. Blue arrows in the image indicate the epithelium layer, orange arrows indicate the dermal gap and black circle indicate the hair follicles; (**G**) Quantitative analyses of the dermal gap during the re-epithelization phase of wound healing; (**H**) Histological analysis on wound tissue sections stained with Masson's trichrome staining after 7 and 14 days of different treatments. Collagen in the image appears in blue, while cell bodies, muscles and keratin appear in red; (**I**) Quantitative analyses of the collagen deposition during the remodeling phase of wound healing. Data are presented as mean ± standard deviation SD with *p < 0.05, **p < 0.01, ***p < 0.001, ****p <0.0001.

Mice with bacteria infected wounds were used to assess the *in vivo* therapeutic performance of KR-12(Q_5_, D_9_). To establish the model, an 8 mm biopsy punch was used to create full-thickness wounds on the dorsal of mice. After infection with *E. coli* for 2 days, mice were randomly divided into the LL-37 treated group, KR-12(Q_5_, D_9_) treated group, and blank control group (**Figure 5A**). LL-37 instead of KR-12 was chosen as a control because it is the original and well-recognized candidate. It has been proven to possess additional therapeutic functions such as modulation of pro-inflammatory response, influence on cell proliferation and differentiation, as well as promotion of wound healing and angiogenesis 56. While these functions have been rarely found for KR-12. Therefore, only LL-37 was set as a control to reduce the number of mice used in experiment. The three selected treatment solutions were applied dropwise onto the wound site at days 0, 3, 7, and 10 (**Figure 5A**). During the 14-day healing period, no obvious fluctuation in body weight was observed for all the groups (*p* > 0.05) (**Figure S17**). Whereas, considerably improved wound closure efficacy was displayed by the KR-12(Q_5_, D_9_) treated group compared to the blank control and LL-37 treated groups (**Figure 5B**). After treatment for 3 days, the infected wound area in the KR-12(Q_5_, D_9_) treated group was smaller than that in the blank control and LL-37 treated groups. These differences became more distinct at day 5, day 7 and day 10 (**Figure 5C**). Even though wounds in the blank control and LL-37 treated groups also recovered at day 14 due to self-healing, they were not epithelialized completely as the KR-12(Q_5_, D_9_) treated wounds. Simulation analysis of the infected wound closure progression was visually illustrated in **Figure 5D** for the different treatments.

Besides, during the early healing stage, wounds in the blank control group were yellowish with a large amount of exudate, which is obviously different from the LL-37 and KR-12(Q_5_, D_9_) treated wounds that were relatively cleaner with less exudate (**Figure 5B**). These results suggested the treatment with LL-37 and KR-12(Q_5_, D_9_) could inhibit the growth of bacteria and thus accelerate the infected wound healing. In this case, the antibacterial performance of LL-37 and KR-12(Q_5_, D_9_) was assessed by comparing the CFU of *E. coli* in the infected wounds. As demonstrated in **Figure 5E**, both LL-37 and KR-12(Q_5_, D_9_) could considerably decrease the bacterial burden in the wounds at day 7. However, the LL-37 was less effective than KR-12(Q_5_, D_9_) and no obvious difference was observed between it and the blank control group at day 14.

Wound healing is a continuous process that is composed of hemostasis, inflammation, proliferation and remodeling phases 57, 58. Re-epithelialization is the hallmark of the proliferation phase, indicating wound regeneration. Therefore, the re-epithelialization of each group was compared by histological analysis based on the hematoxylin and eosin (H&E) staining. As shown in **Figure 5F**, both the blank control and LL-37 treated groups displayed incomplete epidermal layers with markedly tissue damage at day 7. By contrast, the KR-12(Q_5_, D_9_) treated group presented an intact epidermal layer (blue arrows), indicating the faster re-epithelialization. At day 14, epidermal layers were formed in all the three groups. However, the three groups had varying degrees of dermal gaps (**Figure 5G**) and only the KR-12(Q_5_, D_9_) treated group exhibited a considerable amount of hair follicles (black circles) (**Figure 5F**). The decreased dermal gap and newborn skin appendage observed for the KR-12(Q_5_, D_9_) treated group indicated the maturity of the regenerated tissue.

In addition, collagen plays an important role during the remodeling phase as it crosslinks with fibroblast for wound contraction 57, 59. Thus, the collagen deposition of each group was compared by histological analysis based on the Masson's trichrome staining. As demonstrated in **Figure 5H**, wound tissues in the blank control and LL-37 group showed sparse and disorganized collagen depositions, which appeared in blue, at day 7. Comparatively, the KR-12(Q_5_, D_9_) treated group presented a denser collagen net. With the increase in healing time, organized collagen deposition was observed in all the three groups. While quantitative analysis showed that the KR-12(Q_5_, D_9_) treated group had a higher content of collagen compared to that in the blank control and LL-37 group after treatment for 14 days (**Figure 5I**).

Overall, the stapled KR-12 derivative KR-12(Q_5_, D_9_) exhibited improved therapeutic performance than LL-37 on the mouse model with an infected wound. It could inhibit the bacterial growth more effectively, accelerate the re-epithelialization more rapidly, and promote the collagen deposition more compactly.

### Anti-inflammatory activity

**Figure 6:**
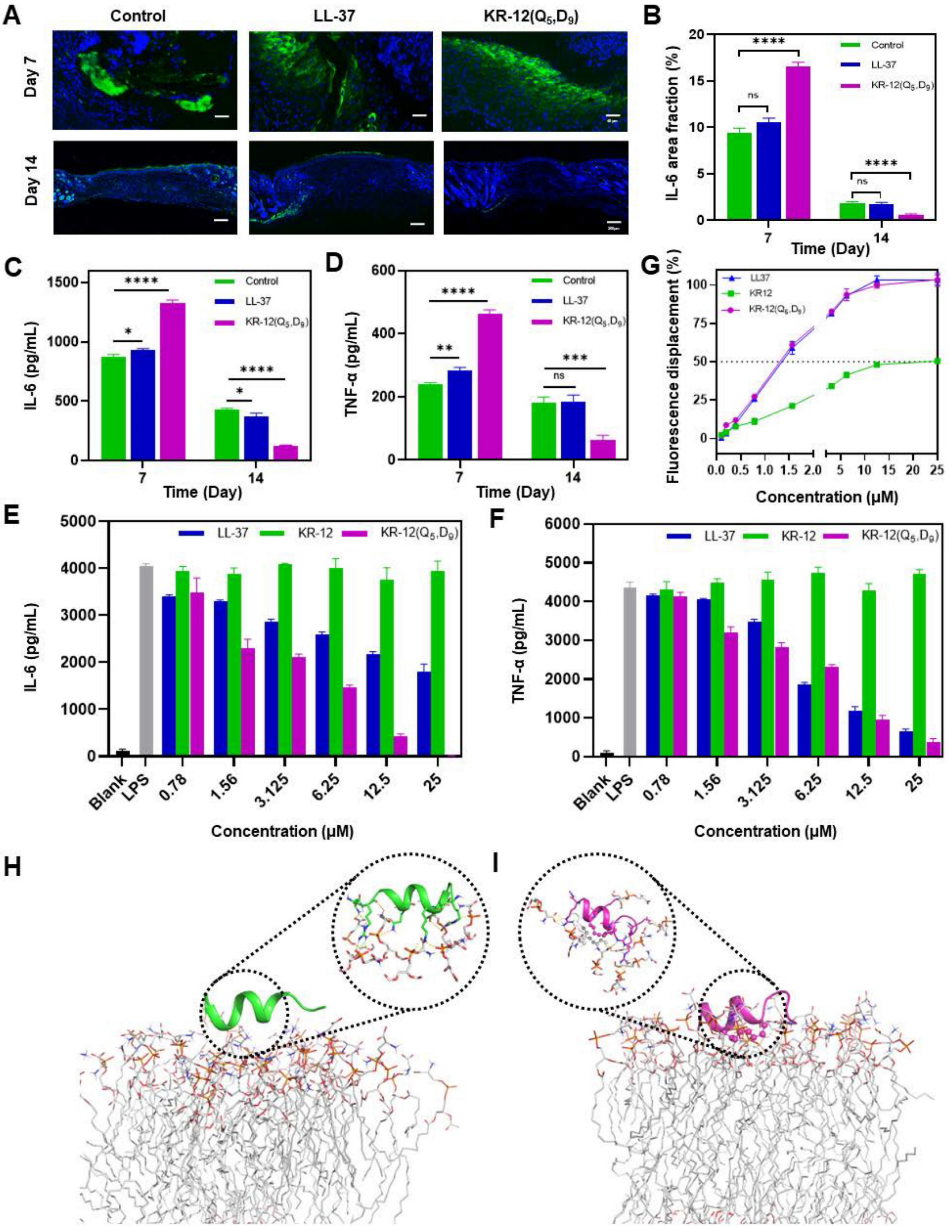
**Anti-inflammatory activity of LL-37 and KR-12(Q_5_, D_9_)**: (**A**) Histological analysis on wound tissue sections stained with immunofluorescence staining after 7 and 14 days of different treatments. IL-6 in the image appears in green; (**B**) Quantitative analyses of the IL-6 during the wound healing; The ELISA determined expression of IL-6 (**C**) and TNF-α (**D**) in the infected wound tissues after 7 and 14 days of different treatments; The ELISA determined secretion of IL-6 (**E**) and TNF-α (**F**) by RAW264.7 cell that stimulated with 200 ng/mL LPS in the presence different concentrations of LL-37 and KR-12(Q_5_, D_9_); (**G**) Fluorescence displacement assay based on the BC probe indicating the LPS affinity capacity of LL-37, KR-12 and KR-12(Q_5_, D_9_). MD simulations of the interactions of KR-12 (**H**) and KR-12(Q_5_, D_9_) (**I**) with the lipid A region of bacterial LPS. The H-bond interactions are indicated by yellow dashed lines. Data are presented as mean ± standard deviation SD with *p < 0.05, **p < 0.01, ***p < 0.001, ****p <0.0001.

To understand the molecular mechanism underlying the improved infected wound healing capacity of KR-12(Q_5_, D_9_), wound tissue from different treatments was histologically analyzed based on immunofluorescence staining to compare the expression level of inflammatory cytokine interleukin-6 (IL-6). IL-6 is secreted by M1 macrophage in response to the stimulation of bacterial lipopolysaccharide (LPS). Its content in tissue is a direct reflection of the level of inflammation. Compared to the blank control and LL-37 treated groups, the KR-12(Q_5_, D_9_) treated group could obviously upregulate the expression of IL-6 at day 7, indicating an acute inflammation status (**Figure 6A**). Whereas, the mostly reduced expression of IL-6 was observed for the KR-12(Q_5_, D_9_) treated group at day 14 (**Figure 6B**), suggesting the acute inflammation was suppressed and switched to the proliferation and remodeling phase. The downregulation of IL-6 was further confirmed by ELISA analysis (**Figure 6C**). Besides, with the KR-12(Q_5_, D_9_) treatment, the same trend of expression, which increased at day 7 and decreased at day 14 was also determined for tumor necrosis factor (TNF-α) (**Figure 6D**). Therefore, it is speculated that KR-12(Q_5_, D_9_) with improved antibacterial activity could eliminate bacteria more effectively at the early stage of wound healing. Upon the lysis of bacteria, the increased amount of bacterial content including LPS could significantly stimulate the macrophage to secrete the inflammatory cytokines, leading to the upregulated expression of IL-6 and TNF-α at day 7. Whereas, the structurally stabilized KR-12(Q_5_, D_9_) may bind to the LPS more compactly, reduce the level of inflammatory cytokines, and thus promote the transition from inflammation to proliferation and remodeling that favors wound healing.

To validate the anti-inflammatory effect of KR-12(Q_5_, D_9_) *in vitro*, RAW264.7 cells were treated with varying concentrations of LL-37, KR-12 and KR-12(Q_5_, D_9_) before the stimulation with 200 ng/mL LPS. As demonstrated in **Figure 6E** and **Figure 6F**, the secretion of both IL-6 and TNF-α was decreased continuously with the increasing concentration of LL-37 and KR-12(Q_5_, D_9_). Notably, the KR-12(Q_5_, D_9_) induced reduction of inflammatory cytokines was more than that induced by LL-37. Whereas, no such effect was observed for KR-12. These results confirmed that the KR-12(Q_5_, D_9_) and LL-37 exerted their anti-inflammatory effects through LPS neutralization. Although the linear KR-12 presented negligible LPS binding capacity, its stapled derivative KR-12(Q_5_, D_9_) acquired such capacity, which was even enhanced than that of LL-37.

To confirm the LPS binding capacity of KR-12(Q_5_, D_9_), a fluorescent displacement assay was further performed utilizing the BODIPY TR cadaverine (BC) probe. The BC probe could bind specifically to the lipid A of LPS and undergo fluorescence self-quenching 60. While its fluorescence would be restored when being competitively displaced by a compound that displays higher affinity.

According to **Figure 6G**, with the increasing concentration of LL-37, KR-12 and KR-12(Q_5_, D_9_), the fluorescence of the BC probe all increased gradually. However, their concentrations that could displace 50% BC varied from 20 μM for KR-12 to approximately 1.3 μM for LL-37 and KR-12(Q_5_, D_9_). Thus, the KR-12(Q_5_, D_9_) was demonstrated to have a similar LPS binding capacity as LL-37, which is better than that of KR-12.

To explain the variance in LPS binding capacity, all-atom MD simulations were performed on the lipid A region of bacterial LPS with the linear KR-12 (**Figure 6H**) and stapled KR-12(Q_5_, D_9_) (**Figure 6I**), respectively. Upon the stapling modification, the all-hydrocarbon staple of KR-12(Q_5_, D_9_) endowed its close interaction with the fatty-acyl chain of lipid A. As a result, more compact H-bonds were formed between the hydrophilic parts of KR-12(Q_5_, D_9_) with the phosphate groups of lipid A molecules in the simulated environment. Therefore, these interaction differences could explain the variance in the LPS neutralization capacity of the linear and stapled KR-12, with the LPS binding capacity of the linear KR-12 being virtually eliminated.

Overall, at the early stage of treatment, KR-12(Q_5_, D_9_) with enhanced antibacterial activity eliminated bacterial more effectively. The increased amount of bacterial content including LPS upon the lysis of bacteria significantly stimulated the acute inflammatory response, leading to the upregulated expression of IL-6 and TNF-α at day 7. Whereas, at the last stage of treatment, the structurally stabilized KR-12(Q_5_, D_9_) binds to LPS more compactly, reducing the level of inflammatory cytokines, and thus promoting the transition from inflammation to proliferation and remodeling phase that favors wound closure and regeneration. Therefore, considerably reduced wound area, rapid re-epithelialization and enhanced collagen deposition were observed at day 14 for the KR-12(Q_5_, D_9_) treatment.

## Conclusion

In conclusion, antimicrobial peptide LL-37 has been recognized as a favorable alternative to antibiotics for the bacterial infection treatment due to its broad antibacterial spectrum, low resistance development and diverse bioactivities including endotoxin neutralizing, immunomodulation, angiogenesis promotion, wound closure *et al* 22-27, 61. However, the high manufactory cost due to long sequence, poor *in vivo* stability against protease hydrolysis, and unpredictable cytotoxicity derived from positive charge have limited its clinical application 28. To push the frontier of clinical application of LL-37, a library of KR-12 derivatives with one- or two-helical wheels stapled at either position of *i, i+4* or *i, i+7* were synthesized and evaluated for antibacterial treatment. Among them, KR-12(Q_5_, D_9_) that stapled at the glutamine and aspartic acid residues displayed both increased positive charge and helical content. The reinforced α-helical conformation of KR-12(Q_5_, D_9_) thereby not only improved its antibacterial and anti-inflammatory efficacy but also protected it from proteolytic hydrolysis. Besides, it also exhibited relatively higher compatibility towards mammalian cells and significantly enhanced therapeutic index. When utilized for the infected wound treatment on mice, it was demonstrated to eliminate bacteria as well as promote wound closure and regeneration effectively. Overall, the all-hydrocarbon stapling of AMP was demonstrated to lay the foundation for the future development of antibacterial agents. The stapled KR-12(Q_5_, D_9_) could serve as a lead compound for the treatment of bacterial infection clinically.

## Supplementary Material

Supplementary materials and methods, figures and table.

## Figures and Tables

**Table 1:** Minimum inhibitory concentration (MIC) of LL-37, KR-12 and its stapled derivatives against a list of gram-positive and gram-negative bacteria.

	Antibacterial activity (MIC in μg mL^-1^)						
	Gram-positive			Gram-negative			
Peptide	*S. aureus*	*S. epidermidis*	*E. faecalis*	*E. coli*	*A. baumannii*	*P. aeruginosa*	*K. pneumoniae*
LL-37	128	> 256	128	128	> 256	> 256	256
KR-12	32	> 256	128	128	> 256	256	> 256
KR-12 (R_2_, R_6_)	128	128	128	>256	> 256	> 256	> 256
KR-12 (I_3_, I_7_)	8	64	16	16	32	256	128
KR-12 (Q_5_, D_9_)	8	16	8	8	16	64	128
KR-12 (I_7_, L_11_)	32	128	32	32	64	256	256
KR-12 (K_8_, R_12_)	16	16	16	64	16	256	> 256
KR-12 (K_1_, K_8_)	> 256	> 256	> 256	>256	> 256	> 256	> 256
KR-12 (R_2_, D_9_)	256	64	256	128	128	256	128
KR-12 (I_3_, F_10_)	64	256	256	64	32	128	64
KR-12 (V_4_, L_11_)	128	256	> 256	128	128	128	128
KR-12 (Q_5_, R_12_)	64	> 256	256	32	64	64	64
PMB^a^	64	128	256	4	4	8	16

^a^ The commercial antibiotic polymyxin b (PMB) was evaluated as control.

**Table 2:** Structural parameters, serum stability and hemolysis of LL-37, KR-12 and its stapled derivatives.

Peptide	T_R_^a^ (min)	NC^b^	α-helicity (%)	T_D50_^c^ (h)	H_256_^d^ (%)
LL-37	15.9	+ 6	32.4 ± 0.5	0.58	9.2 ± 0.2
KR-12	13.7	+ 4	35.0 ± 0.5	0.78	0.6 ± 0.2
KR-12 (R_2_, R_6_)	18.8	+ 2	38.8 ± 0.9	2.38	37.1 ± 0.5
KR-12 (I_3_, I_7_)	14.2	+ 4	39.7 ± 0.8	4.21	0.3 ± 0.1
KR-12 (Q_5_, D_9_)	14.7	+ 5	38.1 ± 0.9	8.91	1.7 ± 0.1
KR-12 (I_7_, L_11_)	14.4	+ 4	42.0 ± 0.8	11.44	1.4 ± 0.1
KR-12 (K_8_, R_12_)	18.2	+ 2	50.0 ± 0.6	18.28	77.2 ± 0.3

^a^Retention time (T_R_) was determined on a reverse-phase C_18_ column based on an elution gradient of CH_3_CN/H_2_O containing 0.1%TFA from 10% to 90% over 20 min; ^b^Net charge (NC) at pH 7.4 was calculated using an online peptide property calculator: http://www.innovagen.com/proteomics-tools; ^c^Degradation time (T_D50_) was defined as the time at which 50% of the peptide is degraded in the presence of serum; ^d^Hemolysis (H_256_) was defined as the hemolysis percentage of rabbit erythrocytes in the presence of 256 μg/mL of LL-37, KR-12 and its stapled derivatives.

## Abbreviations

AMPs: antimicrobial peptides
LPS: lipopolysaccharide
LTA: lipoteichoic acid
S_5_: (S)-2-(4-pentenyl) alanine
R_8_: (R)-2-(7-octenyl) alanine
MIC: minimum inhibitory concentration
CD: circular dichroism
OM: outer membrane
IM: inner membrane
NPN: 1-*N*-phenylnaphthyamine
ONPG: *O*-nitrophenyl-β-D-galactopyranoside
DiSC_3_-5: 3,3′-dipropylthiacarbocyanine iodide
TI: therapeutic index
IL-6: inflammatory cytokine interleukin-6
TNF-α: tumor necrosis factor-α
MD: molecular dynamics
