# Supplementary material for: Discovery of novel antibacterial agent for the infected wound treatment: all-hydrocarbon stapling optimization of LL-37
Source: Theranostics. 2024 Jan 20;14(3):1181–94. doi: 10.7150/thno.87916 (PMC10845205; doi:10.7150/thno.87916)
Supplement: Supplementary file 1 — Supplementary materials and methods, figures and table. [file thnov14p1181s1.pdf]

SUPPLEMENTAL MATERIAL

Discovery of novel antibacterial agent for the infected wound treatment: all-hydrocarbon stapling optimization of LL-37

Yanan Zhang [1,2,#], Mengjun Zheng [3,#], Zhe Wang [4], Zhinan Liu [1,2], Sumeng Chen [1,2], Xiang Li [3,*], Yejiao Shi [1,*], Honggang Hu [2,*]

Affiliations:
[1]Institute of Translational Medicine, Shanghai University, Shanghai 200444, China
[2]School of Medicine, Shanghai University, Shanghai 200444, China
[3]School of Pharmacy, Naval Medical University, Shanghai 200433, China
[4]Institute of Bioengineering, College of Chemical and Biological Engineering, Zhejiang University, Hangzhou, 310027, China

[#]These authors contributed equally to this work.
[*]Corresponding authors.
Yejiao Shi: Email: sh10yj@shu.edu.cn;
Honggang Hu: Email: hhu66@shu.edu.cn

Supplementary Methods

Peptide synthesis and purification

According to our reported method for generating all hydrocarbon stapled peptides, peptides are synthesized using Fmoc-based solid phase peptide synthesis[1]. Using MBHA rink amide resin with a loading capacity of 0.32mmol/g as a solid support, and swelling with DCM for 20 minutes before amino acid coupling cycles. 1: 1: 1 molar ratio of Fmoc-amino acid-OH/ Oxyma pure/ DIC was mixed in NMP for 1 minute and added to the resin for coupling at 60 °C. To obtain different short staple lengths, using two (*S*)-pentenyl alanine residues (S_5) to place α-methyl or α-alkenyl amino acid at positions of *I*, *i+4* or using one (*R*)-octenyl alanine (R_8) at the position of *i* and one S_5 at the position of *i+7*. Fmoc deprotections were carried out twice with 20% piperidine in DMF (v/ v) for 10 minutes. The coupling, washing, deprotection, and washing steps were repeated until all amino acids were conjugated and the *N*-terminus amino groups were acetylated with acetic anhydride/DIEA/DMF for 30 minutes. After acetylation, the stapling reactions were carried out with the Grubbs catalyst first-generation catalyst in DCE for 4 hours. Then use the cleavage cocktail of TFA/water/phenol/TIPs to cut the peptide from the resin at the volume ratio of 88:5:5:2 for 4 hours, while removing all protecting groups Then collect the cleavage solution and remove excess TFA through rotary evaporation. The crude peptide was then precipitated by cold diethyl ether and collected through centrifugation. The crude peptide was rewashed and dried overnight under a vacuum.

The peptide was purified by RP-HPLC (Shimadzu LC-6A, Japan), which was equipped with a preparative RP C18 column (20×250 mm, Daisogel) and gradient of H_2O/ ACN with 0.1% TFA as mobile phase. The fractions containing peptides were collected and their mass was examined by ESI-MS (Shimadzu LCMS-8040, Japan). Concentrate the collected peptide fractions by rotary evaporation, freeze dry, and store at -20 °C.

The mass of the purified peptides was confirmed by ESI-MS (Xevo G2 QTOF, Waters) and the purity of the peptides was analyzed by RP-HPLC (Shimadzu LC-20AD, Japan), equipped with an analytical RP-C18 column (4.6 × 250 mm, Daisogel) and a UV detector set at a wavelength of 220 nm.

Circular dichroism analysis

The secondary structure of the linear and stapled peptides was characterized using a Jasco J-1500 spectropolarimeter (Jasco, Japan) in the far ultraviolet region at 25 °C. Peptide solutions were prepared at 50 μM in pH 7.4 TFE: H_2O at 1:1 (v/v), and loaded into a quartz cuvette with a 1mm path length for measurement. Record the ellipticity (mdeg) signal and convert it to a uniform average residue ellipticity (deg ·

$cm^2 \cdot dmol^{-1}$) after subtracting the background using the following equation: $[\vartheta]=(\vartheta\times1000)/(c\times l\times n)$, among which, ϑ is the ellipticity recorded (mdeg), c is the concentration of peptide (M), l is the quartz cuvette's optical path length (cm), and n is the amino acid number of the peptide. The helicity of peptides was calculated using the following equation: $\alpha = [\vartheta]_{222}/[\vartheta]_{max} \times100\%$, where $[\vartheta]_{222}$ is the mean residue ellipticity of the peptide at 222 nm; $[\vartheta]_{max} = (-44{,}000 + 250T)(1-k/n)$, k = 4, n is the number of amino acids, T = 25 °C.

Bacterial strains and culture

Bacterial type stains ATCC 25923 *S. aureus*, ATCC 12228 *S. epidermidis*, ATCC 29212 *E. faecalis*, ATCC 25922 *E. coli*, ATCC 19606 *A. baumanii*, ATCC 27853 *P. aeruginosa* and ATCC 13883 *K. pneumoniae* were obtained from Shanghai Bioresource Collection Center. The bacterial culture medium was purchased from Oxoid and autoclaved according to the instructions from manufacturer. The isolated strains were cultured in Mueller Hinton II broth and continuously shaken at 140 rpm for 18 hours to the middle of the exponential growth phase at 37 °C with an OD value of 0.4-0.5. Bacterial suspensions with an OD value of 0.1, approximately 10^6 CFU/mL, were then diluted for further assays.

Antibacterial assay

The minimal inhibition concentration (MIC) of the linear and stapled peptides was determined using the microbroth dilution method from the Clinical and Laboratory Standards Institute (CLSI)[2, 3]. Bacterial suspensions of 10^6 CFU/mL were prepared in MH-II broth and peptide solutions at varying concentrations were prepared in phosphate buffer saline (PBS). Then 50 µL peptide solution and 50 µL bacterial suspension were mixed in a 96-well plate. Plates were incubated for 24 hours at 37 °C. 10 µL AlamarBlue cell viability reagent (ThermoFisher Scientific, UK) was added to each well, then the plate was re-incubated for a further 2 hours at 37 °C. The MIC was recorded as the minimum concentration of peptides required to completely inhibit bacteria growth. For serum stability assessment, the peptide solutions were pre-incubated with 50% human serum for 4 h before the MIC determination. Experiments were performed in 3 independent measurements.

Serum stability assay

Serum stability of the linear and stapled peptides was examined by RP-HPLC as previously described[4-6]. 2 mg/mL peptide solution was prepared in pH 7.4 PBS and incubated with human serum (Gemini, USA) to the final volume ratio of 1:4 at 37 °C. At various time intervals, 50 µL mixture was added to 50 µL ethanol to terminate the enzymatic hydrolysis. After centrifugation at 10,000 rpm for 10 minutes under 4 °C,

the supernatant was collected and analyzed by RP-HPLC, with a UV detector monitored at 220 nm. Record the peak area of the intact peptide and calculate the percentage of remaining peptide based on the integrated area as, Peptide remaining (%) = peak area of remaining peptide/peak area of intact peptide×100%. Experiments were performed in 3 independent measurements with the standard error (SD) displayed in the histogram.

Hemolysis assay

0.2 µL of peptide stock solution at the concentration of 256 mg/mL was mixed with 200 µL of 4% (v/v) rabbit erythrocyte suspension, and the mixed solution was diluted with rabbit red blood cell suspension to obtain a series of peptide stock solution with different concentrations by fold dilution. Incubate the plate at 37 °C for 1 hour, then centrifuge at 1000 rpm for 10 minutes. Separate the supernatant and use a microplate reader (BioTek, USA) to measure hemoglobin release. Measure the absorbance at a wavelength of 570nm and calculate the percentage of hemolysis as follows: hemolysis (%) = ($Abs_{peptide}$-Abs_{blank})/($Abs_{control}$-Abs_{blank}), where Abs_{blank} and $Abs_{control}$ were absorbance of samples treated with PBS and 0.1% Triton X-100, respectively.

Outer membrane permeability assay

N-phenyl-1-naphthylamine (NPN, Macklin, China) was used to evaluate the integrity of OM of *E. coli* treated with the linear and stapled peptides. Briefly, overnight cultured bacteria were centrifuged and washed for 3 times before being suspended in 5 mM pH 7.0 HEPES containing 5 mM of glucose to an OD_{600} value of 0.1. Then mix the bacterial suspension with an equal volume of peptide solution in a 96-well plate and incubate for 24 hours at 37 °C. Subsequently, add a final concentration of 10 µM of NPN in each well of the plate and after incubation for 30 minutes, record the fluorescence at 420 nm using a microplate reader with an excitation wavelength of 350 nm. Experiments were performed in 3 independent measurements with SD displayed in the histogram.

Inner membrane depolarization assay

3,3-dipropylthiadicarbocyanine iodide ($DiSC_3(5)$, Aladdin, China) was used to evaluate the degree of IM depolarization of *E. coli* treated with the linear and stapled peptides. Briefly, overnight cultured bacteria were centrifuged and washed for 3 times before being suspended in 5 mM pH 7.0 HEPES containing 5 mM of glucose to an OD_{600} value of 0.1. Then mix the bacterial suspension with an equal volume of peptide solution in a 96-well plate and incubate for 24 hours at 37 °C. Subsequently, add a final concentration of 5 µM of $DiSC_3(5)$ in each well of the plate and after incubation for 30

minutes, record the fluorescence at 670 nm using a microplate reader with an excitation wavelength of 622 nm. Experiments were performed in 3 independent measurements with SD displayed in the histogram.

Extracellular β-galactosidase determination

2-nitrophenyl-β-D-galactopyranoside (ONPG, Sigma, China) was used to evaluate the degree of IM permeability of *E. coli* treated with the linear and stapled peptides. Briefly, the overnight cultured bacteria were centrifuged and washed with PBS for 3 times, and then suspended in 5 mM pH 7.0 HEPES containing 5 mM of glucose to an OD_{600} value of 0.1. The bacterial suspension was then mixed with an equal volume of peptide solution into a 96-well plate and incubated it for 24 hours at 37 °C. Then centrifuge at 3000 rpm for 10 minutes and add 80 μL supernatant to a 96-well plate, each well containing a final concentration of 3 mM of ONPG. Absorbance at 420 nm was recorded after 30 min incubation at 37 °C by a microplate reader. Experiments were performed in 3 independent measurements with SD displayed in the histogram.

Scanning electron microscopy imaging

Bacteria treated with the linear and stapled peptides were centrifuged and washed 3 times with PBS. The obtained bacterial pellets were resuspended in 2.5% glutaraldehyde and incubated it for 24 hours at 4 °C. The bacterial cells were washed three times with PBS and fixed with 1% OsO_4 at 4 °C for 1.5 hours. The fixed bacteria were sequentially dehydrated for 15 minutes by a graded ethanol series at each concentration, with the 100% ethanol dehydration repeated 3 times. The ethanol was then replaced with tert-butyl alcohol for 30 minutes at 4 °C, which was finally removed by a freeze dryer (Labconco, USA). The obtained bacterial powder was coated with platinum and was visualized with a JSM-7800F SEM (JEOL, China).

Live/Dead staining assays

According to the instructions from manufacturer, membrane permeability was measured utilizing the SYTO 9/PI Live/Dead Bacterial Double Staining Kit (Maokang, Shanghai, China). In short, wash the exponential *E. coli* with PBS three times, and then incubate it with the final concentration of 64 μg/mL of peptide at 37 °C for 24 hours. Add SYTO 9 and PI in the commercial reagent kit to the treated cells and stain them for 15 minutes in the dark at room temperature. Stained cells are visualized using **Operetta CLS™high content analysis system** (PerkinElmer, USA), which has excitation/emission wavelengths of 480/500 nm for SYTO 9 and 490/635 nm for PI. Dead bacteria with disrupted membranes were stained with PI, emitting red fluorescence. Whereas, live bacteria with intact membranes were stained with SYTO 9, emitting green fluorescence

Cell lines and culture

Mouse embryonic fibroblast cells NIH-3T3 and mouse monocyte macrophage leukemia cells RAW264.7 were obtained from Shanghai Institute of Biochemistry and Cell Biology, Chinese Academy of Sciences. These two cells were maintained in DMEM and RPMI-1640, respectively. Both media were supplemented with 10% FBS and 1% 100 U/mL penicillin-streptomycin solution. Cells were maintained at 37 °C in a 5% CO_2 and humidified incubator. Replace the culture medium every day until the cell grows to confluence. Cells were then treated with 0.25% trypsin–EDTA and split using fresh medium for either passage or analysis.

Cytotoxicity assay

CCK-8 cell counting kit was utilized to examine the cytotoxicity of the linear and stapled peptides. NIH-3T3 cell was planked at a density of 1.5×10^4 cell/well in a 96-well plate and incubated it for 24 hours at 37 °C. Cells were then treated with different concentrations of peptides in fresh DMEM and incubated for 24 hours before the addition of 10 μL CCK-8 and incubation for a further 1 hour. Measure the absorbance at 450nm using a microplate reader (BioTek, USA) and calculate the percentage of cell viability as: cell viability (%) = $(Abs_{peptide}\text{-}Abs_{blank})/(Abs_{control}\text{-}Abs_{blank})\times100\%$, where $Abs_{control}$ is the absorbance of cells treated with PBS and Abs_{blank} is the absorbance of the cell-free medium. The data was fitted using the nonlinear regression within Graphpad Prism to obtain the IC_{50} values. Experiments were performed in 3 independent measurements with SD displayed in the histogram.

Mouse wound infection model

The animal experiment was approved by the Ethics Committee of Shanghai University (ECSHU). 6- 8 week adult female BALB/c mice with weight of 15-20 g were obtained from Jiangsu Huachuang Xinnuo Pharmaceutical Technology Co. Ltd (Jiangsu, China) and were housed in standard plastic rodent cages under an artificially controlled environment: 50%-70% humidity, 25 °C, and 12 hours light/dark cycle. All mice were anesthetized with 4% chloral hydrate at a dosage of 10 mL/kg with intraperitoneal injection before shaving with a razor. Then a biopsy punch was used to create a full-thickness wound with a diameter of 8mm on the back of the mice and were infected with 20 μL *E. coli* suspension with a density of 2.5×10^8 CFU/mL in pH 7.4 PBS. Cover the infected wound with sterile gauze and fix it with an elastic bandage.

Infected wound healing evaluation

After continuous infection for two days, the mice were divided into 3 groups, 10 in each group, and were treated with 20 μL PBS, 20 μL LL-37 solution (12.8 mg/mL, 100 × MIC), as well as 20 μL KR-12(Q_5, D_9) solution (0.8 mg/mL, 100 × MIC), respectively.

The treatments were repeated on days 0, 3, 7 and 10 and the body weight and wound area of mice were recorded on days -2, 0, 3, 5, 7, 10, 12 and 14. The percentage of wound closure was analyzed and calculated using Image J. The pictured wound area at different time points for each treatment group was simulated and overlaid in Image J to provide direct visualization of the infected wound closure progression. On days 7 and 14, the mice were sacrificed and the wound tissues were collected. The excised tissues were divided into three parts. One part was homogenized in PBS and plated on Luria-Bertani (LB) agar for CFU counting. The second part was fixed overnight with 4% paraformaldehyde, embedded in paraffin and sliced into slices of 5 μm. Hematoxylin-eosin (H&E) staining is used to determine the progress of wound healing, and Masson's trichrome staining is used to analyze collagen deposition. The stained tissue sections were visualized under an ECLIPSE Ni-E microscope (Nikon, Japan). Immunofluorescence staining was used for the analysis of the inflammatory factor IL-6, and the images were obtained through company scanning (Servicebio, China).

Pro-inflammatory cytokine evaluation

First, thaw the rapidly frozen skin tissues and weigh them. Then, add RIPA lysis buffer supplemented with protein inhibitor PMSF (Biyuntian, China) for homogenization. Centrifuge the lysis buffer at 4 °C and 15000 rpm/min for 10 minutes, and transfer the supernatants to clean microcentrifuge tubes. Collect and dilute 20 μL of each supernatant, and measure protein concentration using the BCA method (Biyuntian, China). The expression levels of IL-6 and TNF-α in the tissues were then assessed by the commercially available ELISA kits (Lianke, China) based on the manufacturer's instructions.

Cytokines assay

Seed RAW264.7 cells at 4×10^4 cell/well density in a 48-well plate and incubate it for 24 hours at 37 °C. Then treat the cells with varying concentrations of peptides in fresh RPMI-1640 and incubate for 2 h before the addition of 2 μL LPS (10 μg mL^{-1}) and the incubation for another 6 hours. After 10 minutes of centrifugation at 1800 rpm, the cell supernatants were collected and the expression level of IL-6 and TNF-α was determined by commercially available ELISA kits (Lianke, China) based on the manufacturer's instruction. Experiments were performed in 3 independent measurements with SD displayed in the histogram.

Enzyme-linked immunosorbent assay

Taking IL-6 for example, the enzyme-linked immunosorbent assay (ELISA) of IL-6 was performed in the following steps according to the manufacturer's instructions:

(1) Prepare all reagents and gradient diluted standards. Add 300 μL 1× wash solution to 96-well plates and soak it for 30 seconds; (2) Add 100 μL of twice the diluted standard to the standard well, and add 100 μL of standard dilution solution or culture medium to the blank well; (3) Add sample to the sample well (The specific volume depends on the type of sample); (4) Add 50 μL of 1:100 diluted test antibody to each well; (5) Seal the membrane, incubate at room temperature for 1.5 hours, and wash 6 times; (6) Add 100 μL of 1:100 diluted horseradish peroxidase labeled streptavidin to each well; (7) Seal the membrane, incubate at room temperature for 30 minutes, and wash 6 times; (8) Add 100 μL of chromogenic substrate to each well, keep away from light, and incubate at room temperature for 5-30 minutes; (9) Add 100 μL termination solution to each hole; (10) Within 30 minutes, the OD value is detected at 450 nm wavelength, and the reference wavelength is 570 nm or 630 nm.

LPS binding assay

The LPS binding ability of linear and stapled peptides was evaluated using the displacement method of the BODIPY TR cadaverine (BC) probe reported previously[7]. The BC (Thermo Fisher, D6251) and LPS (Sigma-Aldrich, L2880-25MG) used for measurement were diluted in pH 7.4 Tris-buffer containing 150 mM NaCl and 10 mM Tris to a final concentration of 5 μM and 10 μg/mL, respectively. Add AMPs and LPS probe mixture in Tris-buffer into a 96-well plate and record fluorescence at 620nm by a microplate reader with an excitation wavelength of 580nm after incubation for 1 hour. Experiments were performed in 3 independent measurements with SD displayed in the histogram.

Molecular dynamics (MD) simulations

MD simulations were exploited to study the binding of linear or stapled peptides to lipid A. The initial structures of the peptides were built using ESMFold. Non-standard amino acids in the stapled peptides were parameterized using the GAFF2 force field in Amber. The peptide was built in a membrane environment of lipid A using packmol-memgen. $CaCl_2$ was added to neutralize the system.

MD simulations were carried out using GROMACS 2022.3 with the Amber FF14SB force field. Energy minimization was carried out using the steepest descent algorithm until the maximum force fell below 1000 kJ mol^{-1} nm^{-1}. The system was equilibrated in 6 cycles by releasing position restraints on the peptides and lipids gradually. Periodic boundary conditions were applied. Production runs of 200 ns were performed in the NPT ensemble at 313 K and 1 bar. The temperature was controlled with the Nosé-Hoover thermostat and pressure was controlled using the Parrinello-Rahman barostat. Electrostatic interactions were calculated based on the particle-mesh Ewald method.

The Lennard-Jones potential was utilized for van der Waals interactions. The time step was 2 fs and trajectories were sampled every 10 ps.

Statistical analysis

Experiments were performed in 3 independent measurements. Numerical data was exhibited as mean ± SD. Unless otherwise specified, independent sample t-tests are utilized to calculate the significant differences (*$p \le 0.05$, **$p \le 0.01$, ***$p \le 0.001$, and ****$p \le 0.0001$).

References

1. Li X, Chen S, Zhang WD, Hu HG. Stapled Helical Peptides Bearing Different Anchoring Residues. Chem Rev. 2020; 120: 10079-144.
2. Wojciechowska M, Macyszyn J, Miszkiewicz J, Grzela R, Trylska J. Stapled Anoplin as an Antibacterial Agent. Front Microbiol. 2021; 12: 772038.
3. Shao CX, Jian Q, Li BW, Zhu YJ, Yu WK, Li ZY, et al. Ultrashort All-Hydrocarbon Stapled α-Helix Amphiphile as a Potent and Stable Antimicrobial Compound. J Med Chem. 2023; 66:11414-27.
4. Lu J, Owen SC, Shoichet MS. Stability of Self-Assembled Polymeric Micelles in Serum. Macromolecules. 2011; 44: 6002-8.
5. Zheng MJ, Cong W, Peng HR, Qing J, Shen HX, Tang YX, et al. Stapled Peptides Targeting SARS-CoV-2 Spike Protein HR1 Inhibit the Fusion of Virus to Its Cell Receptor. J Med Chem. 2021; 64: 17486-95.
6. Cong W, Shen H, Liao X, Zheng M, Kong X, Wang Z, et al. Discovery of an orally effective double-stapled peptide for reducing ovariectomy-induced bone loss in mice. Acta Pharm Sin B. 2023; 13: 3770-81.
7. Liao M, Gong H, Quan X, Wang Z, Hu X, Chen Z, et al. Intramembrane Nanoaggregates of Antimicrobial Peptides Play a Vital Role in Bacterial Killing. Small. 2023; 19: e2204428.

Supplementary Figures

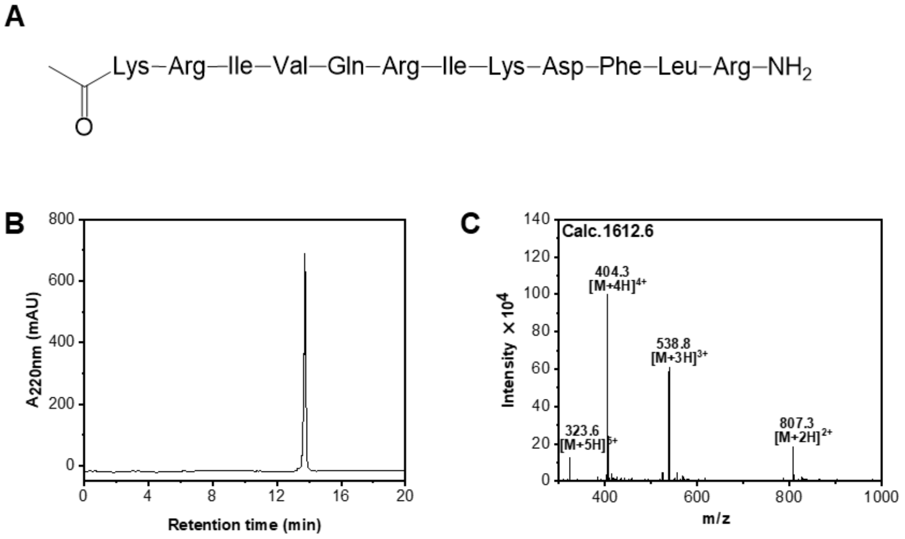

Figure S1. Characterization of KR-12 (Ac-KRIVQRIKDFLR-CONH$_2$): (**A**) Sequence of KR-12; (**B**) analytical RP-HPLC trace under the gradient of 90% to 10% H_2O (10% to 90% ACN) with 0.1% TFA from 2 to 20 min showing high purity; (**C**) ESI-MS spectrum showing the expected molecular mass ($C_{73}H_{129}N_{25}O_{16}$, Mw: 1612.60 g/mol).

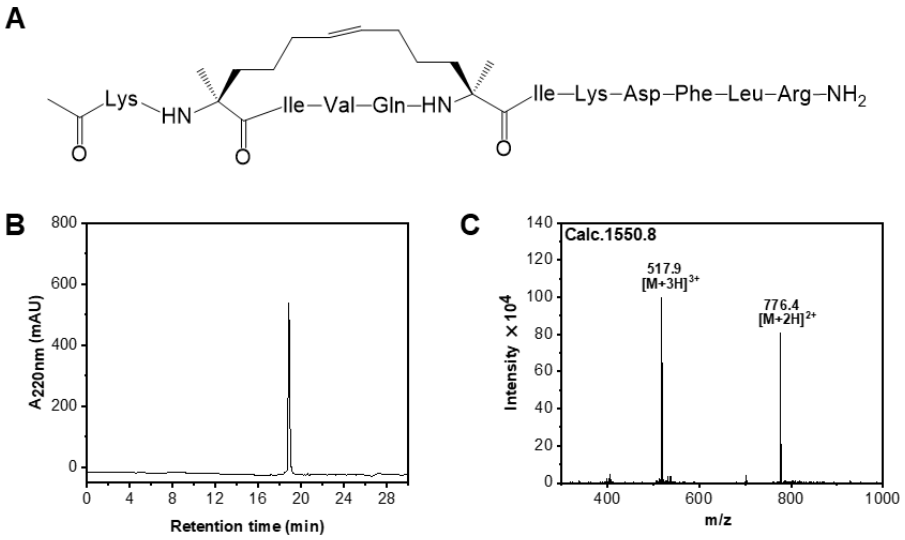

Figure S2. Characterization of KR-12(R$_2$, R$_6$) (Ac-KS$_5$IVQS$_5$IKDFLR-CONH$_2$): (**A**) Sequence of stapled KR-12 derivative; (**B**) analytical RP-HPLC trace under the gradient of 90% to 10% H_2O (10% to 90% ACN) with 0.1% TFA from 2 to 20 min showing high purity; (**C**) ESI-MS spectrum showing the expected molecular mass ($C_{75}H_{127}N_{19}O_{16}$, Mw: 1550.80 g/mol).

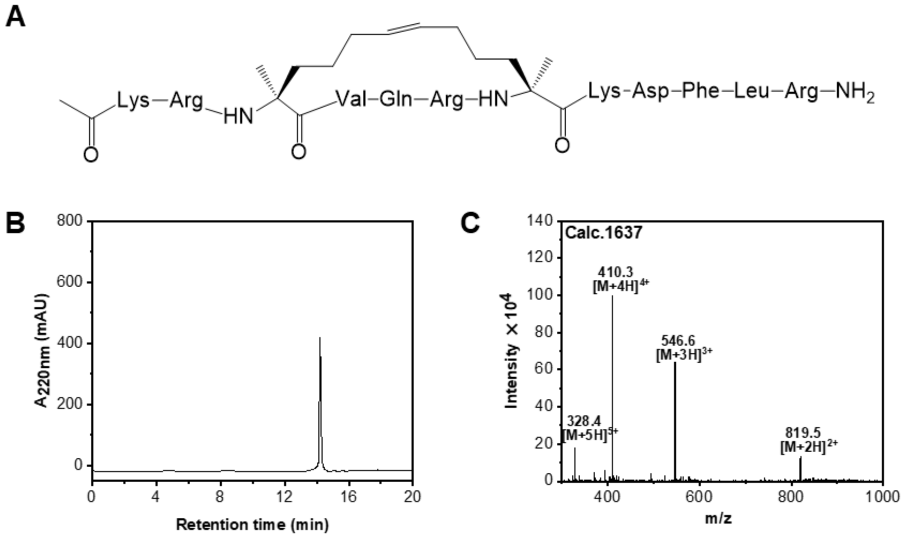

Figure S3. Characterization of KR-12(I_3, I_7) (Ac-KRS$_5$VQRS$_5$KDFLR-CONH$_2$): (**A**) Sequence of stapled KR-12 derivative; (**B**) analytical RP-HPLC trace under the gradient of 90% to 10% H_2O (10% to 90% ACN) with 0.1% TFA from 2 to 20 min showing high purity; (**C**) ESI-MS spectrum showing the expected molecular mass ($C_{75}H_{129}N_{25}O_{16}$, Mw: 1637.00 g/mol).

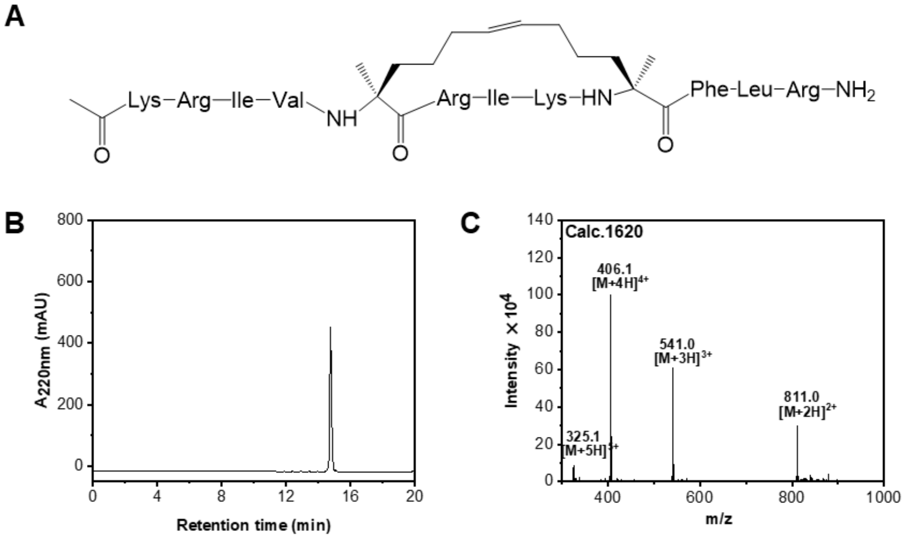

Figure S4. Characterization of KR-12(Q_5, D_9) (Ac-KRIVS$_5$RIKS$_5$FLR-CONH$_2$): (**A**) Sequence of stapled KR-12 derivative; (**B**) analytical RP-HPLC trace under the gradient of 90% to 10% H_2O (10% to 90% ACN) with 0.1% TFA from 2 to 20 min showing high purity; (**C**) ESI-MS spectrum showing the expected molecular mass ($C_{78}H_{138}N_{24}O_{13}$, Mw: 1620.00 g/mol).

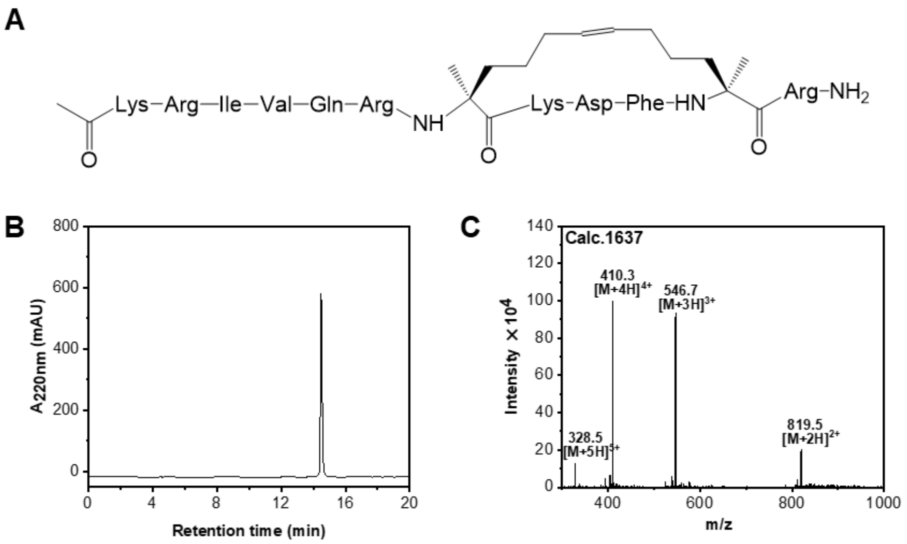

Figure S5. Characterization of KR-12(I_7, L_{11}) (Ac-KRIVQRS$_5$KDFS$_5$R-CONH$_2$): (**A**) Sequence of stapled KR-12 derivative; (**B**) analytical RP-HPLC trace under the gradient of 90% to 10% H_2O (10% to 90% ACN) with 0.1% TFA from 2 to 20 min showing high purity; (**C**) ESI-MS spectrum showing the expected molecular mass ($C_{75}H_{129}N_{25}O_{16}$, Mw: 1637.00 g/mol).

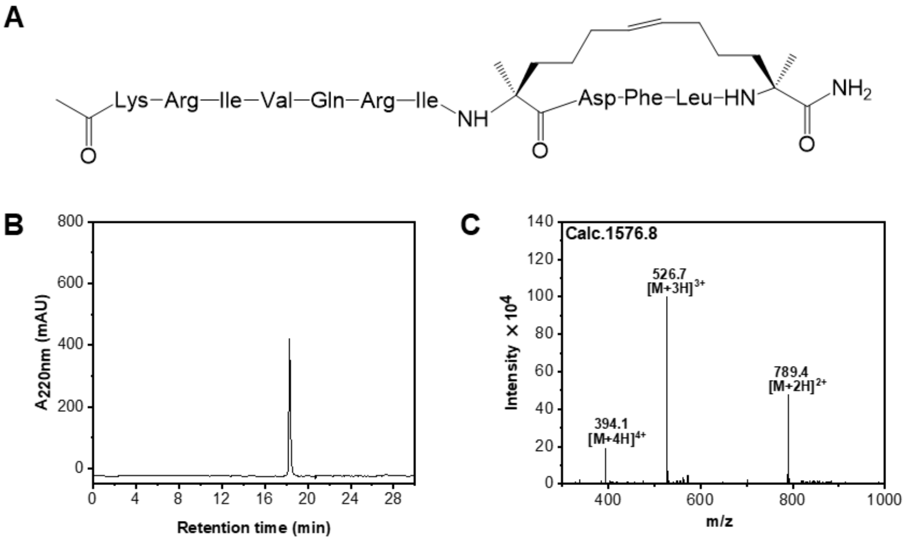

Figure S6. Characterization of KR-12(K_8, R_{12}) (Ac-KRIVQRIS$_5$DFLS$_5$-CONH$_2$): (**A**) Sequence of stapled KR-12 derivative; (**B**) analytical RP-HPLC trace under the gradient of 90% to 10% H_2O (10% to 90% ACN) with 0.1% TFA from 2 to 20 min showing high purity; (**C**) ESI-MS spectrum showing the expected molecular mass ($C_{75}H_{127}N_{21}O_{16}$, Mw: 1576.80 g/mol).

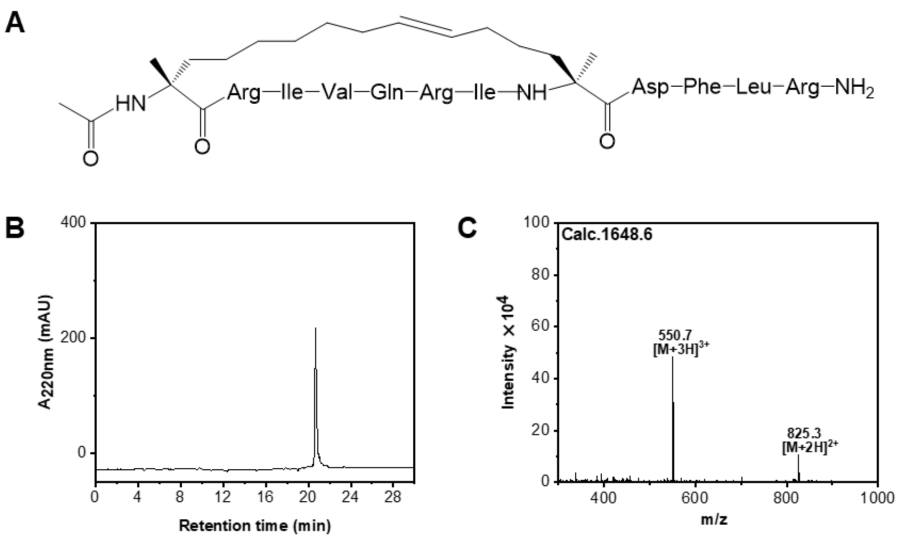

Figure S7. Characterization of KR-12(K$_1$, K$_8$) (Ac-R$_8$RIVQRIS$_5$DFLR-CONH$_2$): (**A**) Sequence of stapled KR-12 derivative; (**B**) analytical RP-HPLC trace under the gradient of 90% to 10% H_2O (10% to 90% ACN) with 0.1% TFA from 2 to 20 min showing high purity; (**C**) ESI-MS spectrum showing the expected molecular mass ($C_{78}H_{133}N_{23}O_{16}$, Mw: 1648.60 g/mol).

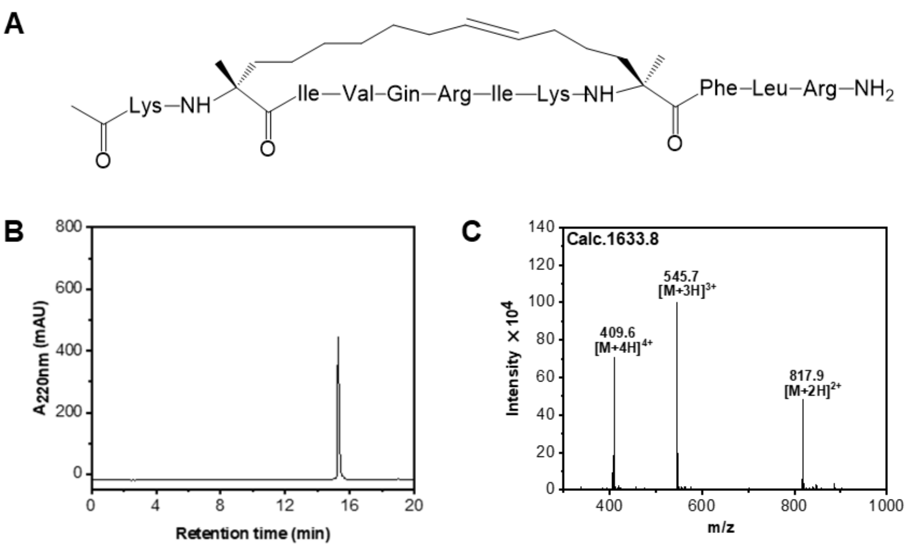

Figure S8. Characterization of KR-12(R$_2$, D$_9$) (Ac-KR$_8$IVQRIKS$_5$FLR-CONH$_2$): (**A**) Sequence of stapled KR-12 derivative; (**B**) analytical RP-HPLC trace under the gradient of 90% to 10% H_2O (10% to 90% ACN) with 0.1% TFA from 2 to 20 min showing high purity; (**C**) ESI-MS spectrum showing the expected molecular mass ($C_{80}H_{140}N_{22}O_{14}$, Mw: 1633.80 g/mol).

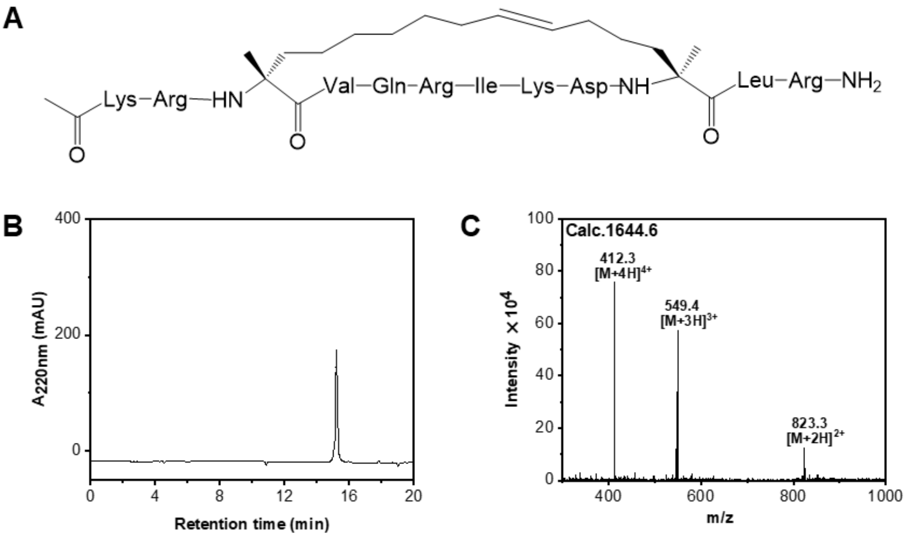

Figure S9. Characterization of KR-12(I$_3$, F$_{10}$) (Ac-KRR$_8$VQRIKDS$_5$LR-CONH$_2$): (**A**) Sequence of stapled KR-12 derivative; (**B**) analytical RP-HPLC trace under the gradient of 90% to 10% H_2O (10% to 90% ACN) with 0.1% TFA from 2 to 20 min showing high purity; (**C**) ESI-MS spectrum showing the expected molecular mass ($C_{75}H_{137}N_{25}O_{16}$, Mw: 1644.60 g/mol).

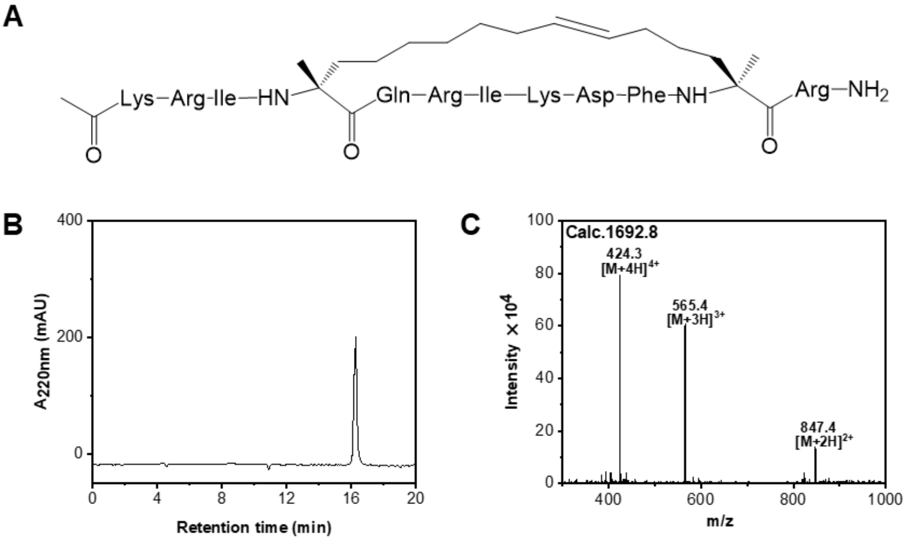

Figure S10. Characterization of KR-12(V$_4$, L$_{11}$) (Ac-KRIR$_8$QRIKDFS$_5$R-CONH$_2$): (**A**) Sequence of stapled KR-12 derivative; (**B**) analytical RP-HPLC trace under the gradient of 90% to 10% H_2O (10% to 90% ACN) with 0.1% TFA from 2 to 20 min showing high purity; (**C**) ESI-MS spectrum showing the expected molecular mass ($C_{79}H_{137}N_{25}O_{16}$, Mw: 1692.80 g/mol).

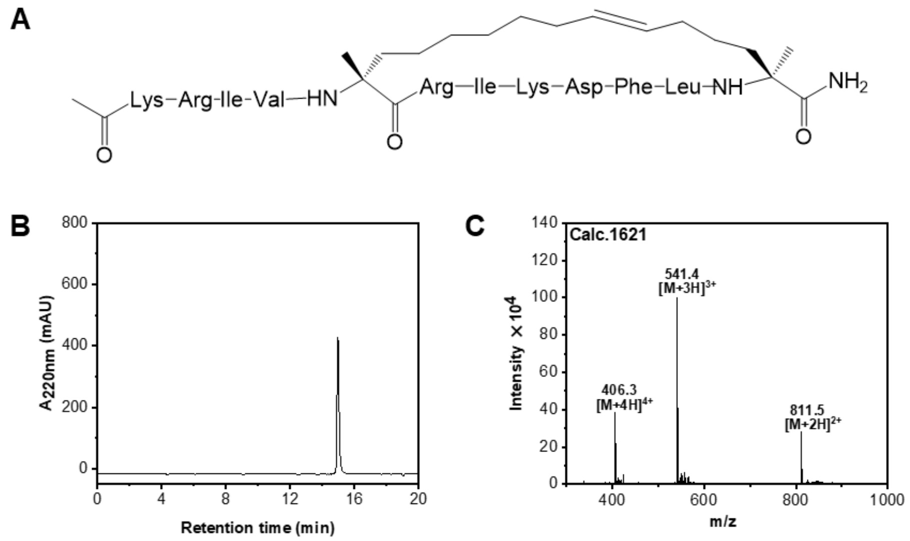

Figure S11. Characterization of KR-12(Q_5, R_{12}) (Ac-KRIVR$_8$RIKDFLS$_5$-CONH$_2$): (**A**) Sequence of stapled KR-12 derivative; (**B**) analytical RP-HPLC trace under the gradient of 90% to 10% H_2O (10% to 90% ACN) with 0.1% TFA from 2 to 20 min showing high purity; (**C**) ESI-MS spectrum showing the expected molecular mass ($C_{79}H_{137}N_{21}O_{15}$, Mw: 1621.00 g/mol).

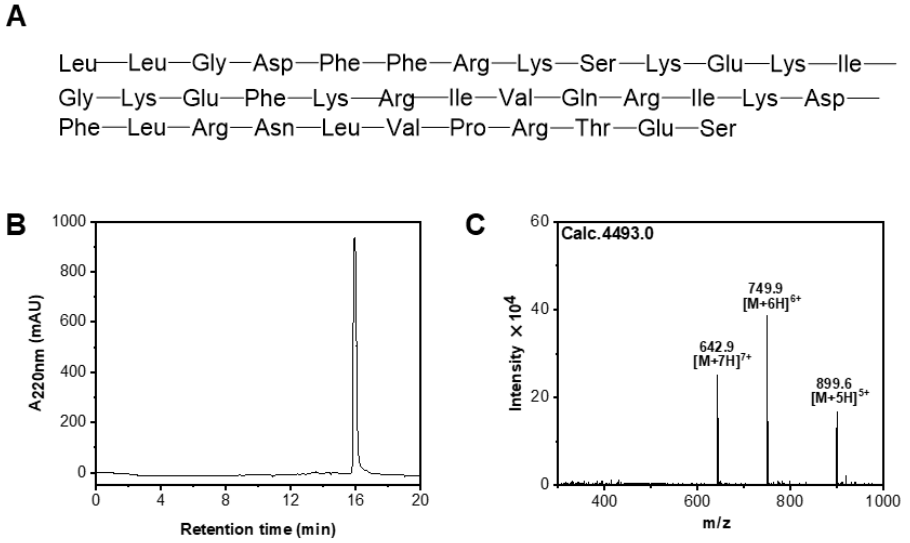

Figure S12. Characterization of LL-37 (COOH-LLGDFFRKSKEKIGKEFKRIVQRIKDFLRNLVPRTES-CONH$_2$): (**A**) Sequence of LL-37; (**B**) analytical RP-HPLC trace under the gradient of 90% to 10% H_2O (10% to 90% ACN) with 0.1% TFA from 2 to 20 min showing high purity; (**C**) ESI-MS spectrum showing the expected molecular mass ($C_{205}H_{340}N_{60}O_{53}$, Mw: 4493.00 g/mol).

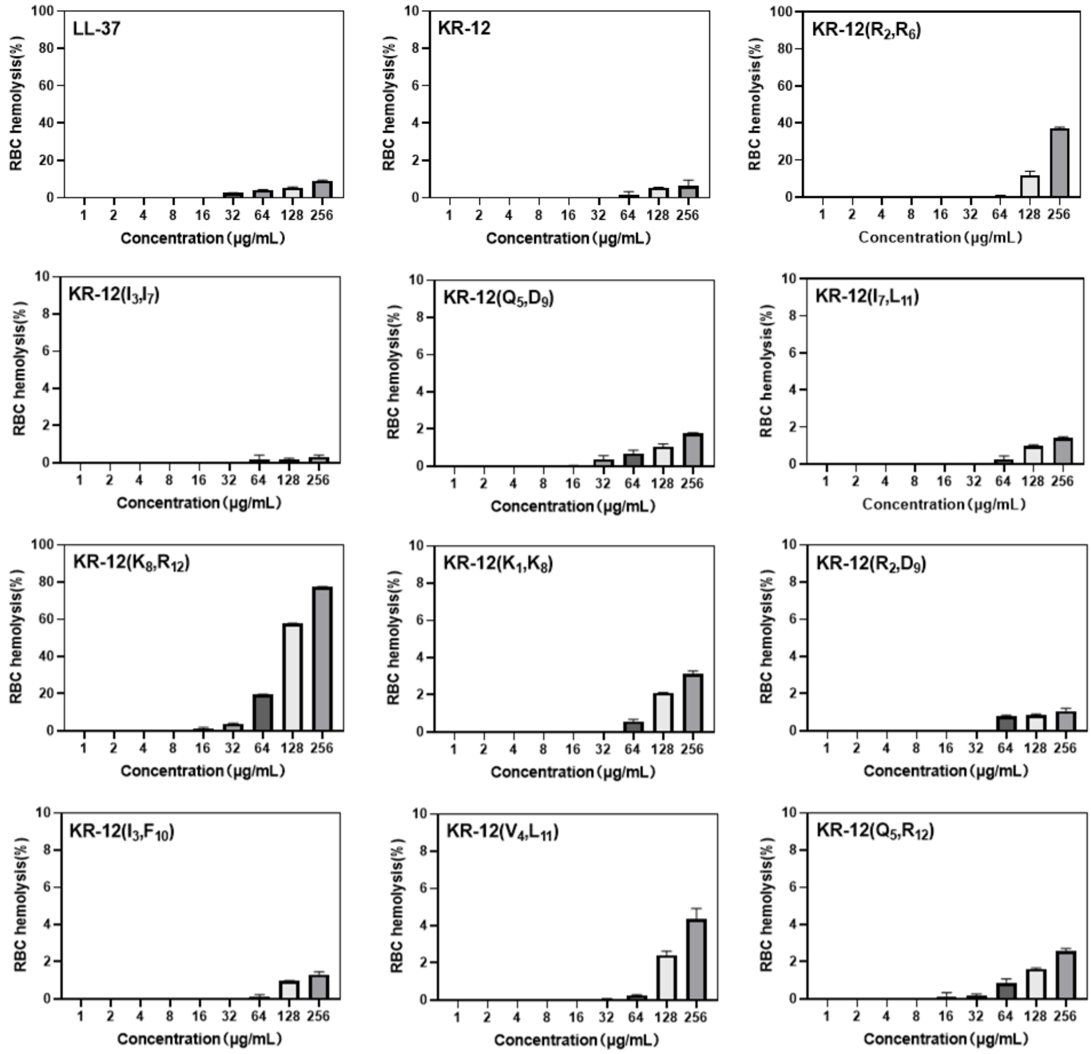

Figure S13. Hemolysis of LL-37, KR-12 and its stapled derivatives towards rabbit erythrocyte.

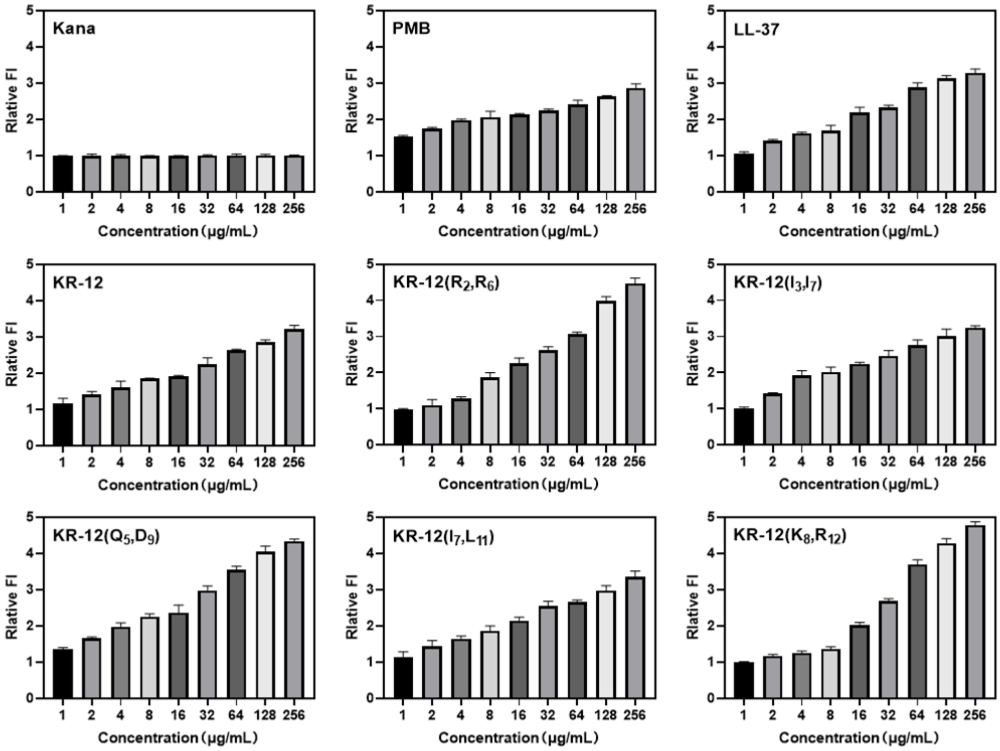

Figure S14. Outer membrane perturbation assay performed with NPN.

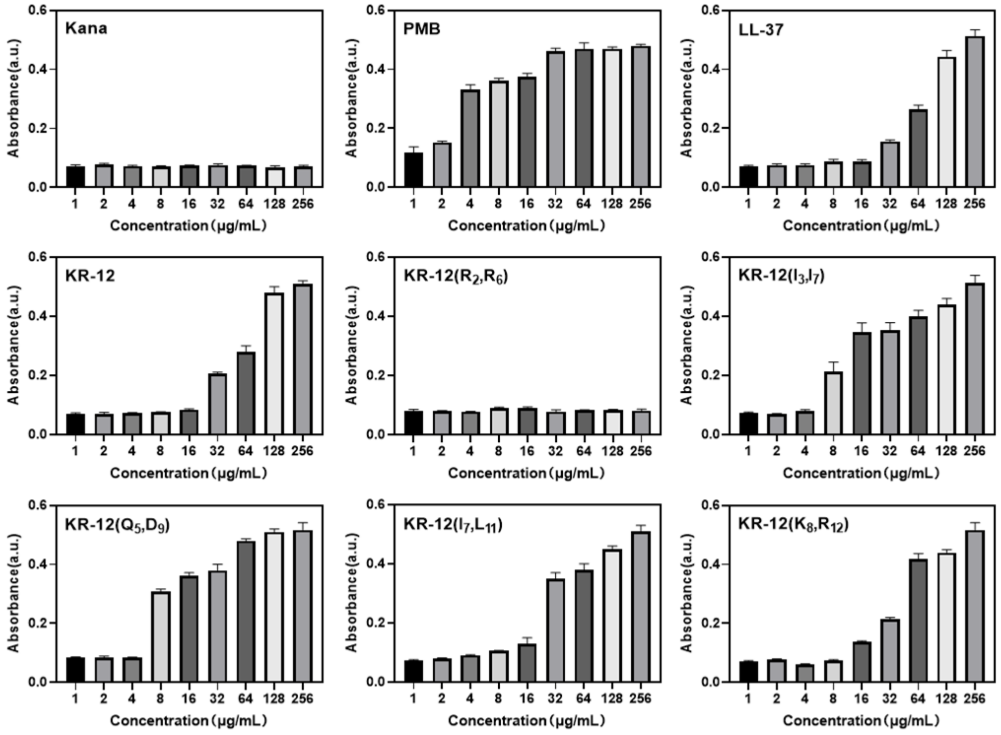

Figure S15. Inner membrane permeability assay performed with ONPG.

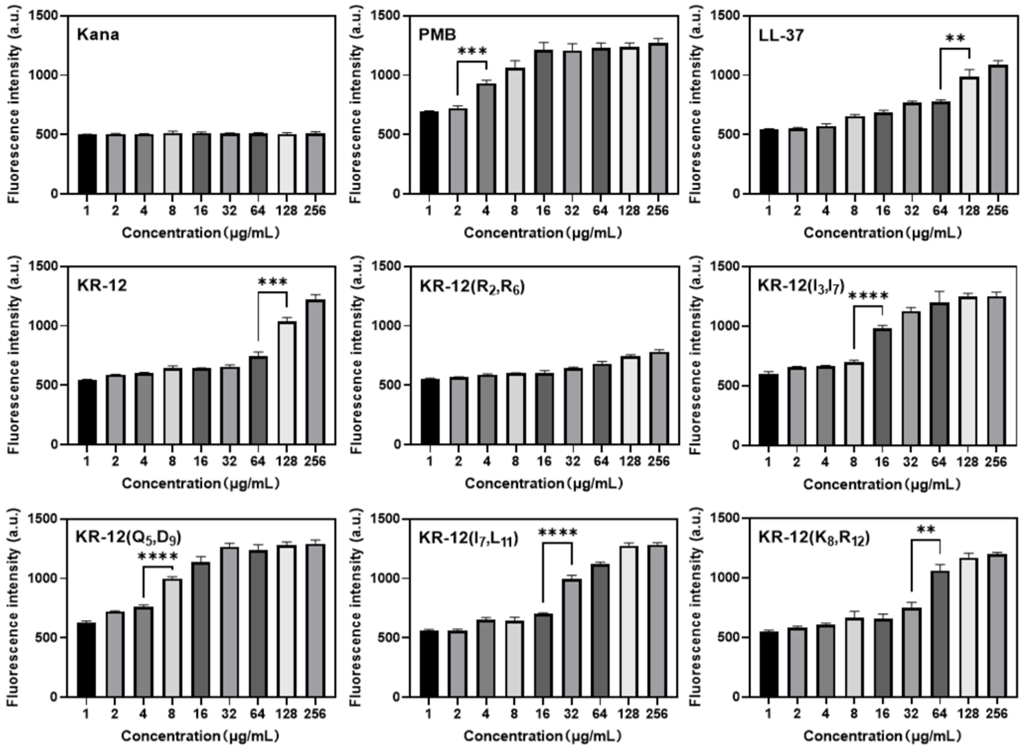

Figure S16. Inner membrane depolarization assay performed with DiSC$_3$-5.

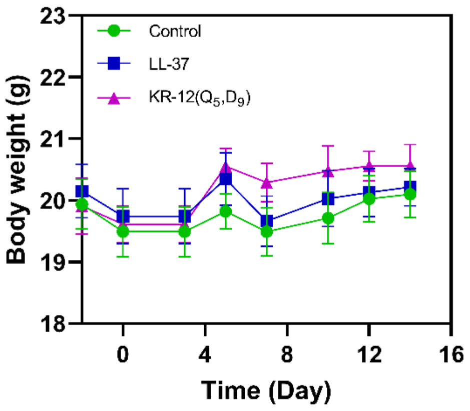

Figure S17. Body weight of mice with the different treatments for 14 days.

Table S1. Minimum inhibitory concentration (MIC) of LL-37, KR-12 and KR-12(Q_5, D_9) against a list of gram-positive and gram-negative bacteria before pre-incubation with 50% human serum for 4 h.

	Antibacterial activity (MIC in µg mL^{-1})						
	Gram-positive			Gram-negative			
**Peptide**	***S. aureus***	***S. epidermidis***	***E. faecalis***	***E. coli***	***A. baumannii***	***P. aeruginosa***	***K. pneumoniae***
LL-37	>256	>256	>256	128	>256	>256	>256
KR-12	>256	>256	>256	>256	>256	>256	>256
KR-12(Q_5, D_9)	8	8	16	8	16	64	128
